# Coronin 1C restricts endosomal branched actin to organize ER contact and endosome fission

**DOI:** 10.1083/jcb.202110089

**Published:** 2022-07-08

**Authors:** Jonathan F. Striepen, Gia K. Voeltz

**Affiliations:** 1 Department of Molecular, Cellular, and Developmental Biology, University of Colorado, Boulder, CO; 2 Howard Hughes Medical Institute, Chevy Chase, MD

## Abstract

ER contact sites define the position of endosome bud fission during actin-dependent cargo sorting. Disrupting endosomal actin structures prevents retrograde cargo movement; however, how actin affects ER contact site formation and endosome fission is not known. Here we show that in contrast with the WASH complex, actin, its nucleator ARP2/3, and COR1C form a contained structure at the bud neck that defines the site of bud fission. We found that actin confinement is facilitated by type I coronins. Depletion of type I coronins allows actin to extend along the length of the bud in an ARP2/3-dependent manner. We demonstrate that extension of branched actin prevents ER recruitment and stalls buds before fission. Finally, our structure-function studies show that the COR1C’s coiled-coil domain is sufficient to restore actin confinement, ER recruitment, and endosome fission. Together, our data reveal how the dynamics of endosomal actin and activity of actin regulators organize ER-associated bud fission.

## Teaser

ER contact sites define the position of endosome bud fission during actin-dependent cargo sorting. Striepen Voeltz show that COR1C confines actin at endosome bud necks. Depleting COR1C and its paralogs causes ARP2/3-dependent actin extension along the bud, resulting in loss of ER contact, and blocks endosome fission.

## Introduction

The endocytic system consists of many independently trafficking vesicles. These vesicles deliver a diverse set of endocytosed cargos to disparate destinations in the cell. Individual endosomes frequently contain heterogeneous cargos that need to be conveyed to different parts of the cell. Endosome fission is a process whereby the endosome segregates cargos that need to be recycled into a bud and then splits off that bud. This process enables endosomes to selectively divert cargos from the terminal lysosome fate, allowing for proper and efficient cargo delivery.

The endosome fission process begins with cargo adaptor complexes such as the retromer and retriever. These complexes recognize different recycling cargos, concentrate the cargo, and recruit downstream factors essential for bud formation and fission (McNally et al., 2017; Seaman, 1998; Harbour et al., 2012; Kvainickas et al., 2017a). Cargo adaptors then bind the Wiskott-Aldrich syndrome protein and scar homologue (WASH) complex, which subsequently binds and activates the branched actin nucleator actin-related protein 2/3 (ARP2/3; Harbour et al., 2012; Simonetti and Cullen, 2019). In turn, ARP2/3 recruits branched F-actin to generate a branched actin structure on the endosome bud (Gomez and Billadeau, 2009; Kvainickas et al., 2017b). This actin structure is proposed to use its membrane remodeling activities to facilitate bud formation and stabilization, cargo binding, and fission (Derivery et al., 2009; Hong et al., 2015; Puthenveedu et al., 2010; Duleh and Welch, 2010; Dong et al., 2016).

Although many and sometimes contradictory membrane remodeling functions are ascribed to actin at the endosome bud, actin dynamics during fission have not been measured directly. Instead, actin’s function at the endosome bud has been studied by disruption of actin assembly and downstream measurements of either retrograde cargo flow or general endosome shape (Duleh and Welch, 2010; Rottner et al., 2010; Simonetti and Cullen, 2019; Puthenveedu et al., 2010). Current models of actin function at endosome fission rely on these endpoint assays and actin membrane remodeling activities characterized at other intracellular membranes (Mayor and Köster, 2016). Without basic measurements of actin dynamics and regulation during fission, its function during this essential step in cargo recycling remains obscure.

The ER has emerged as a principal regulator of the endocytic system through the formation of membrane contact sites (MCSs). Via MCSs, the ER is able to regulate a variety of basic endosome functions and characteristics, including trafficking on microtubules, lipid composition, maturation, and fission (Wu et al., 2018; Wu and Voeltz, 2021; Rocha et al., 2009; Raiborg et al., 2015; Wilhelm et al., 2017; Allison et al., 2017; Dong et al., 2016). We previously identified two factors important for MCS formation and subsequent fission of late endosome (LE) buds marked by the WASH complex: the ER-localized protein TMCC1 and the endosome-localized actin regulatory protein coronin 1C (COR1C; Hoyer et al., 2018).

COR1C and its paralogs in the type I coronin family, COR1A and COR1B, are regulatory hubs of branched actin dynamics (Chan et al., 2011). COR1B and COR1C coimmunoprecipitate with ARP2/3 and the branched actin cleavage protein Cofilin (Cai et al., 2005; Rosentreter et al., 2007). In particular, COR1B ARP2/3 interaction was demonstrated to inhibit ARP2/3 activity (Cai et al., 2007). This regulatory axis between type I coronins and ARP2/3 could be relevant to endosome cargo sorting and fission. ARP2/3 stabilization by Cortactin stabilizes endosome buds to allow slow-diffusing cargos to accumulate in the bud (Puthenveedu et al., 2010). Although fission was not directly examined, these data suggest that bud-localized actin structures might slow or prevent fission and must be disassembled from the endosome bud for fission to occur. If actin clearance is required, it is unknown by what mechanism and to what extent actin would be rearranged. COR1C is a particularly attractive candidate for this function because it colocalizes with actin on endosome buds and, as mentioned before, is required for efficient fission of WASH complex-associated buds (Hoyer et al., 2018; Puthenveedu et al., 2010).

Here we set out to capture a more complete view of branched F-actin dynamics at endosome budding domains and understand the function of COR1C on these structures during the entire endosome bud fission process. We demonstrate that actin is contained to the endosome bud neck by COR1C. Without COR1C and its paralogs, actin begins to proliferate along the length of the endosome bud, preventing proper recruitment of ER MCSs and endosome fission. We show that this regulation is likely achieved by inhibition of ARP2/3 via COR1C’s coiled coil (CC).

## Results

### Coronin and actin regulatory factors are partitioned to the base of the bud during fission

**Figure 1.**
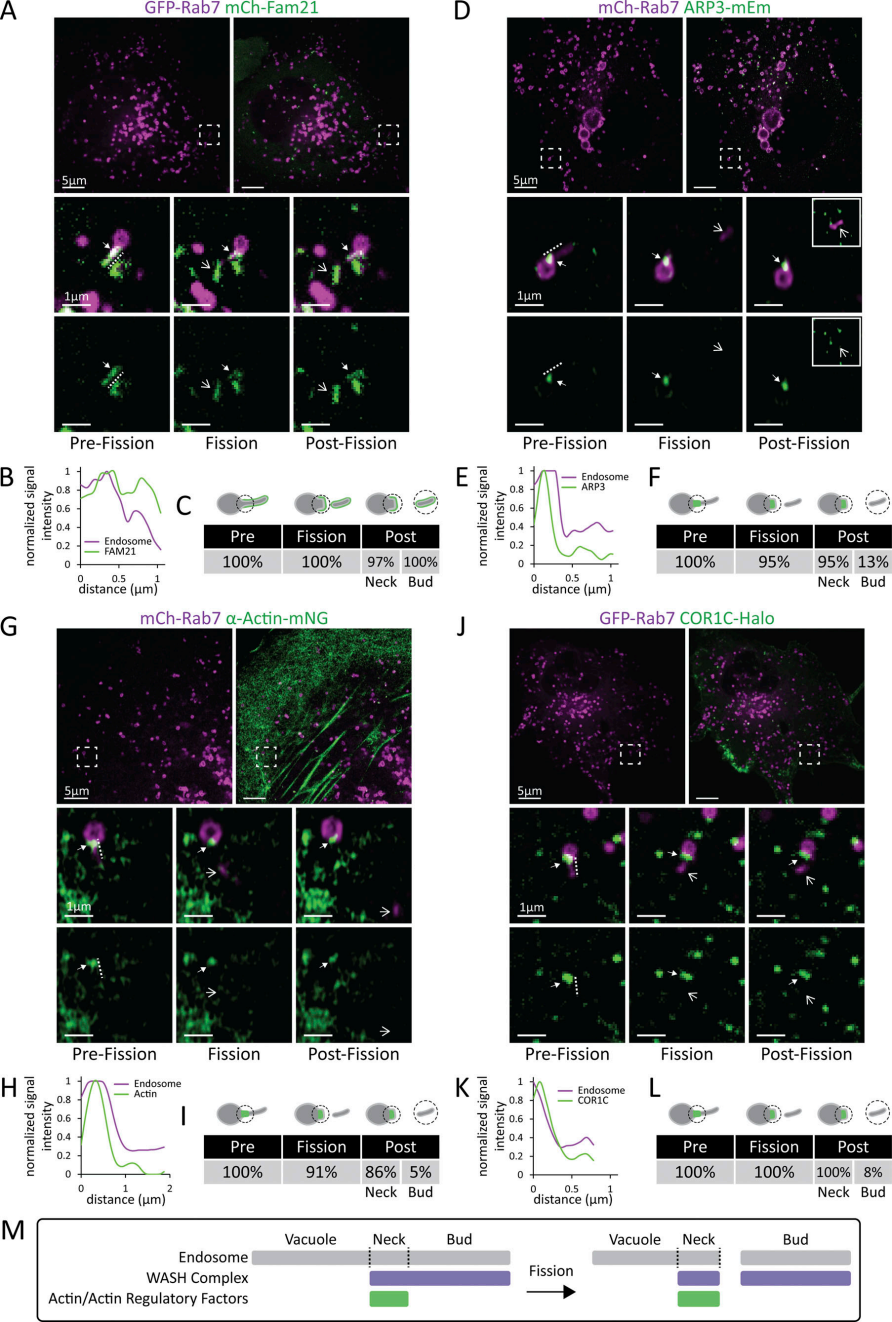
**Coronin and actin regulatory factors are partitioned to the base of the bud during fission. (A)** Representative images of COS-7 cells transfected with GFP-Rab7 (LE, magenta) and mCh-FAM21 (WASH complex, green). Magnified inset (5 × 5 μm) below shows time lapse of a representative fission event. Note: FAM21 signal localizes along entire length of bud, and signal is present on the post-fission bud. Block arrowhead indicates the bud neck, and line arrowhead indicates departing bud (Video 1). **(B)** Line scan analysis along the length of endosome bud in pre-fission frame from A shows FAM21 signal labels the length of the bud. Lines are shown in A adjacent to actual area measured so as not to obscure ROI. **(C)** Fission events as in A were scored for FAM21 enrichment at the bud neck in the pre-fission, fission, and post-fission frames. For post-fission frames, both the bud neck and departed bud were scored for positive FAM21 signal. Table shows data as percentage of fission events with enrichment at each stage (*n* = 37 fission events in 16 cells, performed in triplicate). Model indicates where enrichment was assessed at each stage of fission. **(D–F)** As in A–C, for COS-7 cells transfected with mCh-Rab7 (LE, magenta) and ARP3-mEm (ARP2/3 complex, green). Secondary inset shows departed bud that moved out of primary inset. In F, *n* = 24 fission events in 11 cells, performed in triplicate (Video 2). **(G–I)** As in A–C, for COS-7 cells transfected with mCh-Rab7 (LE, magenta) and α-actin-mNG (actin structure, green). For I, *n* = 19 fission events in 14 cells, performed in triplicate (Video 3). **(J–L)** As in A–C, for COS-7 cells transfected with GFP-Rab7 (LE, magenta) and COR1C Halo (green). For L, *n* = 36 fission events in 21 cells, performed in triplicate. Scale bars for whole cell = 5 μm; insets = 1 μm (Video 4). **(M)** Summary diagram of how actin recruitment and regulatory factors divide during fission.

As a first step toward understanding the function of COR1C during bud fission, we characterized the dynamics and distribution of actin and actin regulatory components before, during, and after endosome bud fission. We cotransfected COS-7 cells with GFP-Rab7 (or mCh-Rab7, to label LEs) and mCh-FAM21 (a component of the WASH complex), ARP3-mEm (ARP2/3 complex), COR1C-Halo, or α-actin-mNG (fluorescently tagged nanobody against actin). We visualized endosome bud fission by live time-lapse confocal fluorescence microscopy (2-min videos at 2-s intervals). Marker distribution was analyzed along the bud at three key reference points: in the pre-fission frame, the fission frame, and the post-fission frame (Fig. 1). These data revealed the relative distributions of these actin regulators and their general dynamics during bud fission. FAM21 (WASH) localized along the entire length of the bud in the pre-fission frame and was split between the endosome vacuole and the bud upon fission (Fig. 1, A and B; and Video 1). In stark contrast, the branched actin nucleator ARP3 (ARP2/3) and actin itself segregated to a small punctum at the base of the endosome bud before and during fission. Neither ARP3 nor actin signal left with the bud post-fission (Fig. 1, D–I; and Videos 2 and 3). This localization is demonstrated by comparing the line scan analyses along the vacuole and bud of the pre-fission frames; only the FAM21 signal enriches along the entire length of the line scan (compare Fig. 1, B, E, and H). COR1C had the same distribution as ARP3 and actin before and after bud fission (Fig. 1, J–L; and Video 4). The data collected were tabulated as the percentage of fission events that had signal at the base of the bud before, during, and after fission. Additionally, for events that had stable signal enrichment at the bud neck post-fission, we measured the percentage of events with signal enrichment leaving with the bud (Fig. 1, C, F, I, and L). This revealed that FAM21 was the only signal consistently departing with the bud. Taken together, our results show that actin structures are maintained at the bud neck throughout the fission process and do not need to be cleared entirely for fission to occur. These data suggest that fission occurs distal to actin and in an actin-free zone, because the signals for actin, ARP2/3, and COR1C segregate on the vacuolar side of the fission site and are rarely found in association with the leaving bud (see summary diagram in Fig. 1 M).

**Video 1.** **FAM21 (green) LE (magenta) fission event from Fig. 1 A.** COS-7 cell transfected with GFP-Rab7 (LE, magenta) and mCh-FAM21 (WASH complex, green) imaged every 2 s. Playback 2 frames/s. First arrow indicates marked bud pre-fission, following two sets of arrows indicate bud neck and departed bud. Note signal localizes along entire bud and leaves with departing bud.

**Video 2.** **ARP3 (green) LE (magenta) fission event from Fig. 1 D.** COS-7 cell transfected with mCh-Rab7 (LE, magenta) and ARP3-mEm (ARP2/3 complex, green) imaged every 2 s. Playback 2 frames/s. First arrow indicates marked bud pre-fission, following two sets of arrows indicate bud neck and departed bud. Note signal localizes only to bud neck and remains behind on the vacuole side.

**Video 3.** **Actin (green) LE (magenta) fission event from Fig. 1 G.** COS-7 cell transfected with mCh-Rab7 (LE, magenta) and α-actin-mNG (actin, green) imaged every 2 s. Playback 2 frames/s. First arrow indicates marked bud pre-fission, following two sets of arrows indicate bud neck and departed bud. Note signal localizes only to bud neck and remains behind on the vacuole side.

**Video 4.** **COR1C (green) LE (magenta) fission event from Fig. 1 J.** COS-7 cell transfected with GFP-Rab7 (LE, magenta) and COR1C-Halo (green) imaged every 2 s. Playback 2 frames/s. First arrow indicates marked bud pre-fission, following two sets of arrows indicate bud neck and departed bud. Note signal localizes only to bud neck and remains behind on the vacuole side.

### Type I coronins confine actin to the bud neck

**Figure 2.**
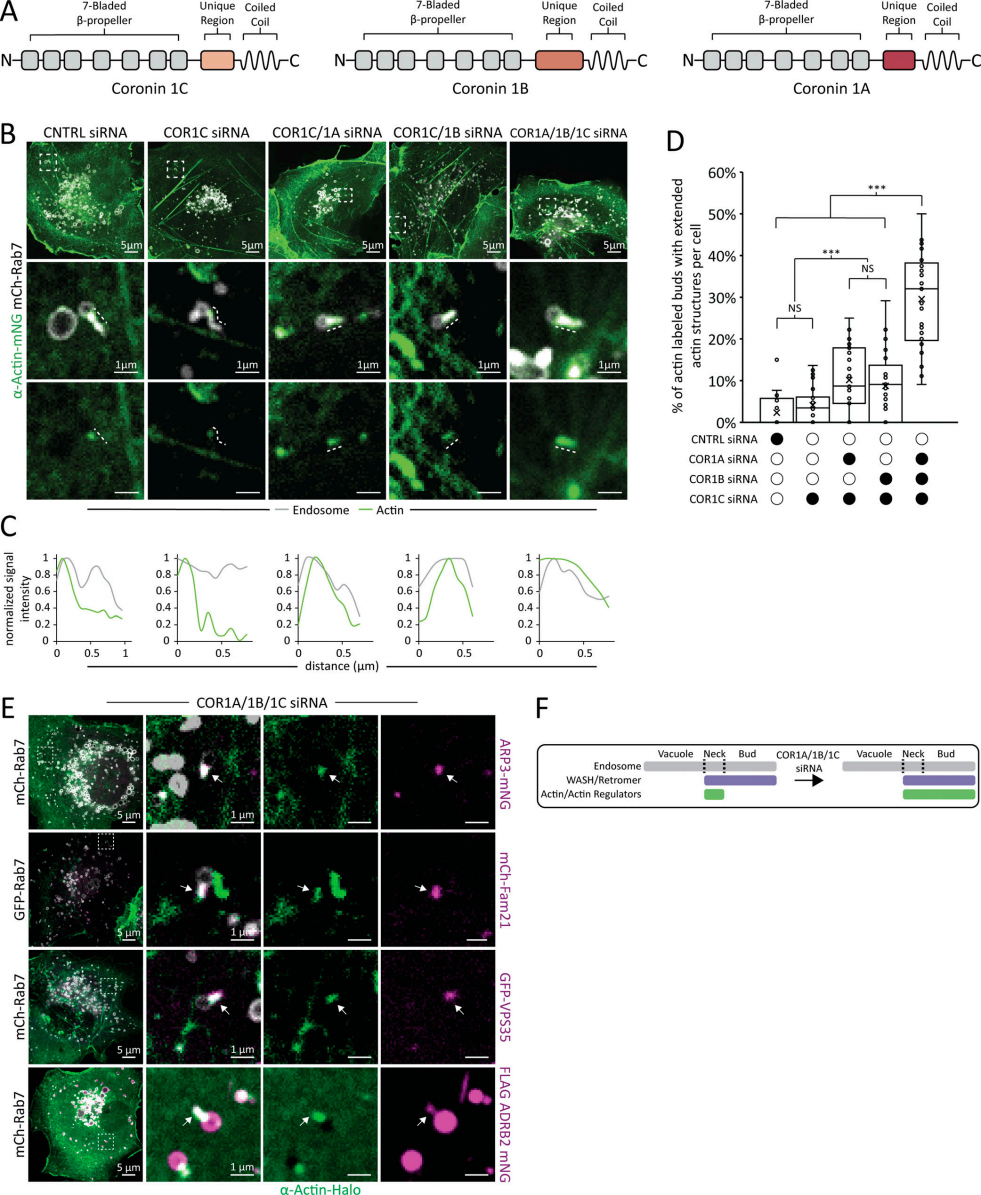
**Type I coronins confine actin to the bud neck. (A)** Domain diagrams of type I coronins showing clear conservation of domain structure indicating the possibility of redundant function. **(B)** Representative images of COS-7 cells transfected with mCh-Rab7 (LE, gray), α actin-mNG (green), and control siRNAs, COR1C siRNAs, COR1C/1A siRNAs, COR1C/1B siRNAs, or COR1A/1B/1C siRNAs. Magnified inset (5 × 5 μm) below shows actin distribution on LE buds. Lines are shown adjacent to actual area measured in B so as not to obscure ROI. Note the extension of actin structures along the distal bud with the simultaneous depletion of two or more type I coronins (Videos 5 and 6). **(C)** Line scan analysis of signal distribution along the bud length for magnified inset examples shown in B. Lines are shown adjacent to actual area measured to not obscure area of interest. Note, actin fluorescent signal spreads into the matching bud signal as more type I coronins are depleted. **(D)** Quantification of data in B. Graph of percentage of actin-labeled buds with extended actin structures in cells treated with either control siRNAs (for 550 endosomes in *n* = 32 cells), COR1C siRNAs (for 598 endosomes in *n* = 23 cells), COR1C/1A siRNA (for 668 endosomes in *n* = 31 cells), COR1C/1B siRNA (for 721 endosomes in *n* = 29 cells), or COR1A/1B/1C siRNAs (for 578 endosomes in *n* = 29 cells), performed in triplicate. X indicates mean, and line indicates median. **(E)** Representative images of COS-7 cells transfected with mCh-Rab7 or GFP Rab7 (LE, gray), α actin-Halo (green), COR1A/1B/1C siRNAs, and with ARP3-mNG (ARP2/3 complex, magenta), mCh-FAM21 (WASH complex, magenta), GFP-VPS35 (retromer complex, magenta), or FLAG-ARDRB2-mNG (membrane cargo, magenta) reveals that the extended actin structures do not disrupt the recruitment of upstream cargo-sorting complexes or sorting of cargo into bud. Arrows indicate endosome bud. Statistical analyses were performed with one-way ANOVA, P value from Tukey’s test: ***, P < 0.001. Scale bars for whole cell = 5 μm; insets = 1 μm. **(F)** Summary figure showing changes in relative localization of actin and cargo-sorting components along the endosome.

**Figure S1.**
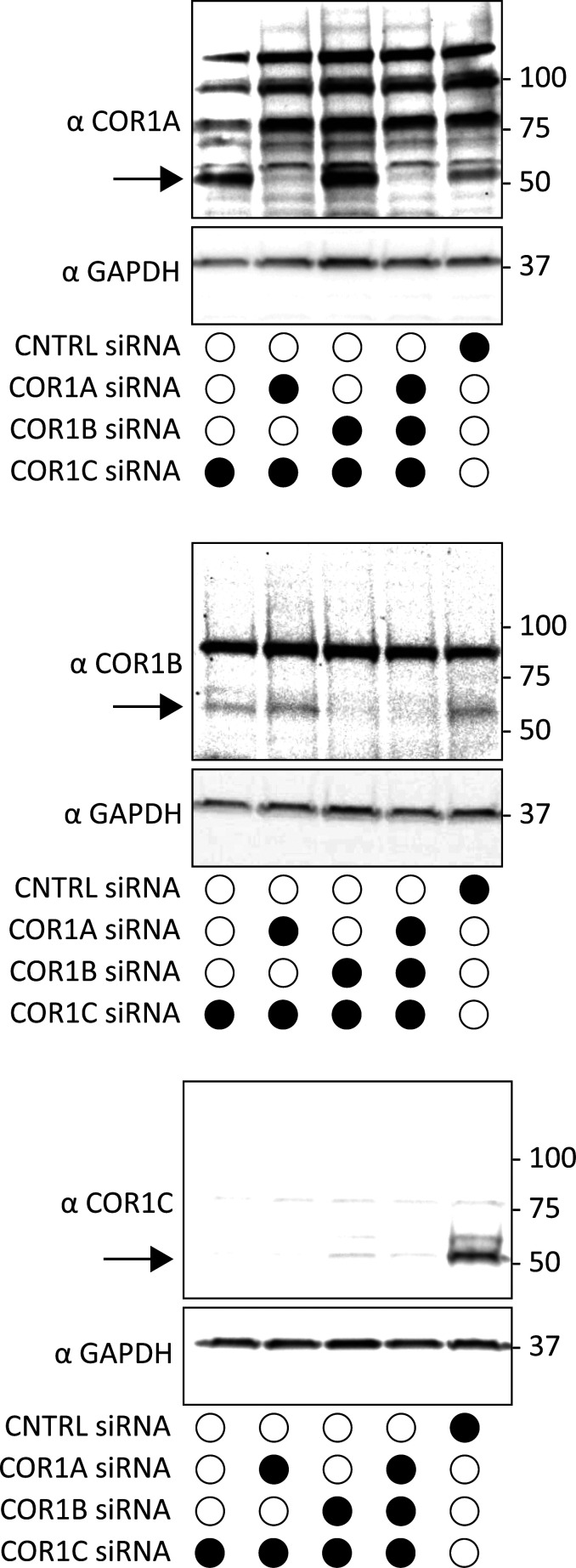
**Immunoblot of type I coronin depletion in COS-7 cells.** Immunoblots for combination depletions tested in Fig. 2 A. Blots show the same samples run three separate times to blot for COR1A, COR1B, and COR1C. Data show that type I coronins can be depleted efficiently individually or in combinations. Source data are available for this figure: SourceData FS1.

Our data demonstrate that a component of the WASH complex localizes along the entire length of the bud, whereas actin, ARP2/3, and COR1C are contained to the base of the bud neck (Fig. 1). Coronins are actin and ARP2/3 binding proteins that in other circumstances (at the cell cortex) have been shown to influence actin turnover by debranching F-actin (Cai et al., 2008; Chan et al., 2011; Cai et al., 2007). We thus asked whether COR1C might function to similarly destabilize ARP2/3 and prevent actin polymerization onto the distal part of the endosome bud. To test this, we depleted COR1C from cells and asked if there was an increase in actin signal along the bud. We cotransfected COS-7 cells with mCh-Rab7 (LEs), α-actin-mNG, and either control or COR1C siRNAs and imaged the cells live. COR1C depletion alone was not sufficient to alter actin localization on the bud compared with control (Fig. 2 B, columns 1 and 2, and Video 5). There are, however, three type I coronin paralogs (COR1A, COR1B, and COR1C). These paralogs have a well-conserved domain structure, and there is evidence indicating they can interact with one another (Huttlin et al., 2021; Chan et al., 2011; Fig. 2 A). We reasoned that in the absence of COR1C, COR1A and COR1B might function redundantly to contain actin to LE bud necks. To deplete combinations of type I coronin proteins, we cotransfected COS-7 cells with mCh-Rab7 (LEs), α-actin-mNG, and either control siRNAs, COR1C siRNAs, COR1C/1A siRNAs, COR1C/1B siRNAs, or COR1A/1B/1C siRNAs. All type I coronins were efficiently depleted under these conditions (Fig. S1). We imaged live cells and collected time-lapse videos to visualize actin patches relative to the endosome buds. When the type I coronins were depleted in pairs, we began to observe actin structures extending along the length of the endosome bud (Fig. 2 B, columns 1 and 2 vs. 3 and 4). When all three type I coronins were depleted from cells, we scored a marked increase in the number of endosomes with these extended actin buds (Fig. 2 B, column 5, and Video 6). This synergistic effect is shown clearly in the line scans along the bud, where actin signal increasingly mirrors bud signal as more type I coronins are depleted (Fig. 2 C). This contrasts with the sharp drop-off in actin signal on the distal bud seen in control or COR1C siRNA treatments (Fig. 2, B and C). We quantified the percentage of actin-labeled buds with extended actin per cell and calculated a mean percentage under each condition (Fig. 2 D). These data revealed a significant increase in actin localization to the distal bud upon depletion of two or more COR1 paralogs and demonstrate that type I coronins can function redundantly to clear actin from the distal part of the endosome bud.

**Video 5.** **Actin (green) localization on LE bud (gray) in control siRNA-treated cell, related to Fig. 2, A and B.** COS-7 cell transfected with mCh-Rab7 (LE, magenta), α-actin-mNG (actin, green), and control siRNAs imaged every 2 s. Playback 4 frames/s. Arrow at paused frame indicates actin marked bud. Note actin confinement to the bud neck.

**Video 6.** **Actin (green) localization on LE bud (gray) in COR1A/1B/1C-depleted cell, related to Fig. 2 B.** COS-7 cell transfected with mCh-Rab7 (LE, magenta), α-actin-mNG (actin, green), and COR1A/1B/1C siRNAs imaged every 2 s. Playback 4 frames/s. Arrows at paused frame indicate actin marked buds. Note actin extension along the bud neck.

Notably, the actin extension on the bud caused by Cor1A/1B/1C depletion did not disrupt the recruitment of upstream components required for bud formation/cargo sorting or the sorting of a model membrane cargo, ADRB2, into the bud. We cotransfected COS-7 cells with COR1A/1B/1C siRNAs to deplete all three coronins; α-actin-Halo to score actin extension; mCh-Rab7 (or GFP-Rab7) to label LEs and ARP3-mNG, mCh-FAM21 (WASH complex), GFP-VPS35 (retromer complex), or FLAG-ADRB2-mNG (membrane cargo). We imaged all markers in live cells and observed efficient enrichment to endosome buds despite the presence of extended actin structures. This supports the idea that these buds are still capable of sorting cargo (Fig. 2 E and summary diagram, Fig. 2 F).

### Type I coronin depletion can be rescued by COR1C

**Figure S2.**
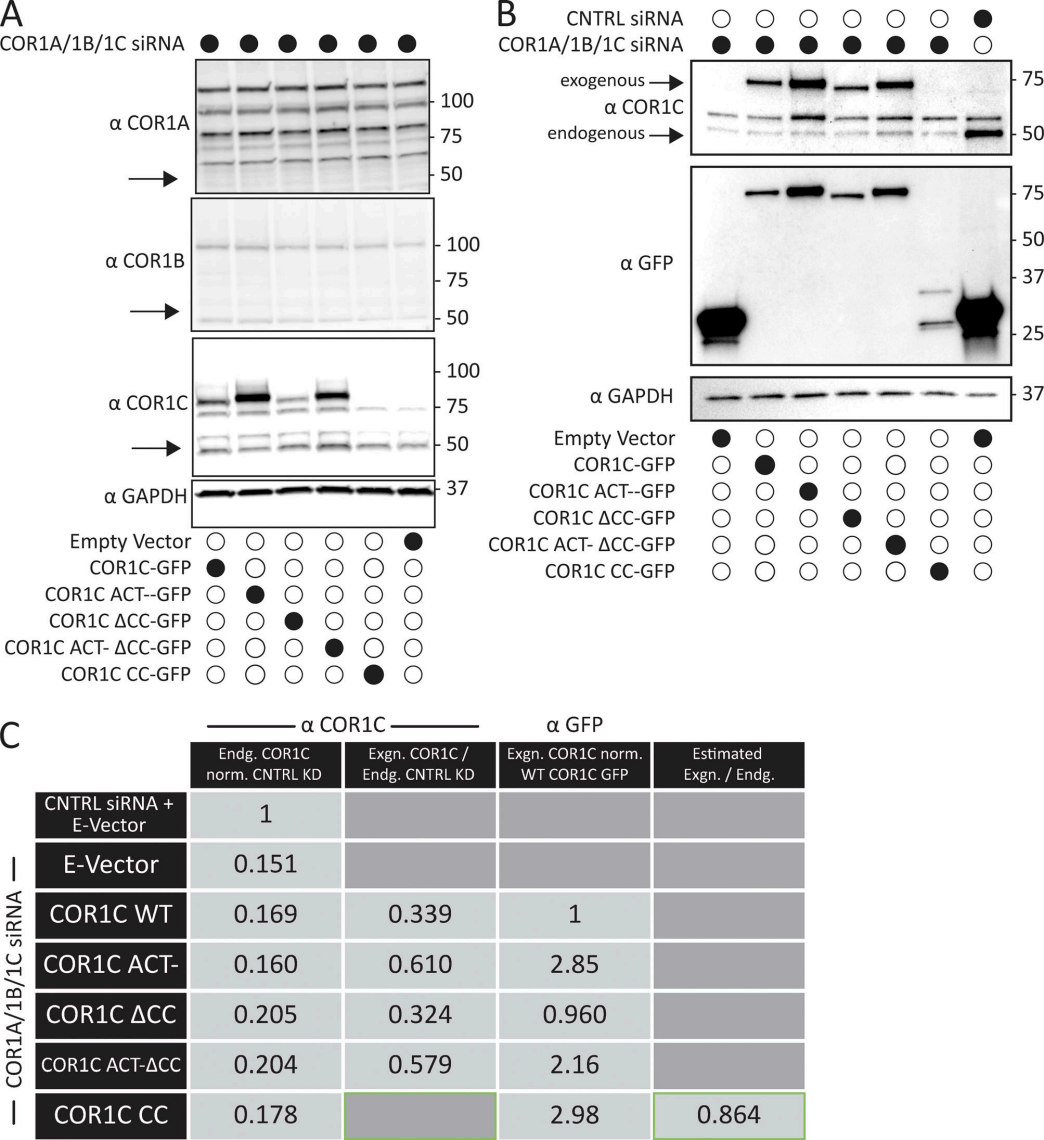
**Immunoblot of type I coronin depletion and rescue in COS-7 cells. (A)** Representative immunoblots for type I coronin depletion (COR1A, COR1B, and COR1C) and rescue as in Fig. 3, B and D. Data show that type I coronins can be depleted efficiently and that the siRES COR1C constructs express well and at comparable levels for rescue (analysis was performed in triplicate). **(B)** Immunoblots for type I coronin depletion and rescue as in Fig. 5 showing relative expression of rescue constructs and endogenous COR1C. Representative immunoblots probed with antibody to COR1C, GFP, and GAPDH. **(C)** Quantification of rescue blots in B. Table shows average normalized ratios from three replicates (blots). Values were calculated as indicated in column headers. Briefly, the first column demonstrates clear KD. The second column demonstrates that expression is comparable between exogenously expressed mutants and is also comparable with endogenous. The third column demonstrates that the relative exogenous rescue expression is comparable to WT rescue. The final column is an estimate of CC expression relative to endogenous COR1C based on the average ratio of anti-GFP to anti-COR1C signal, suggesting that the CC is also not expressed above endogenous. This was necessary because the CC is not detectable via the anti-COR1C antibody and so could not be probed for in the same blot. Source data are available for this figure: SourceData FS2.

**Figure 3.**
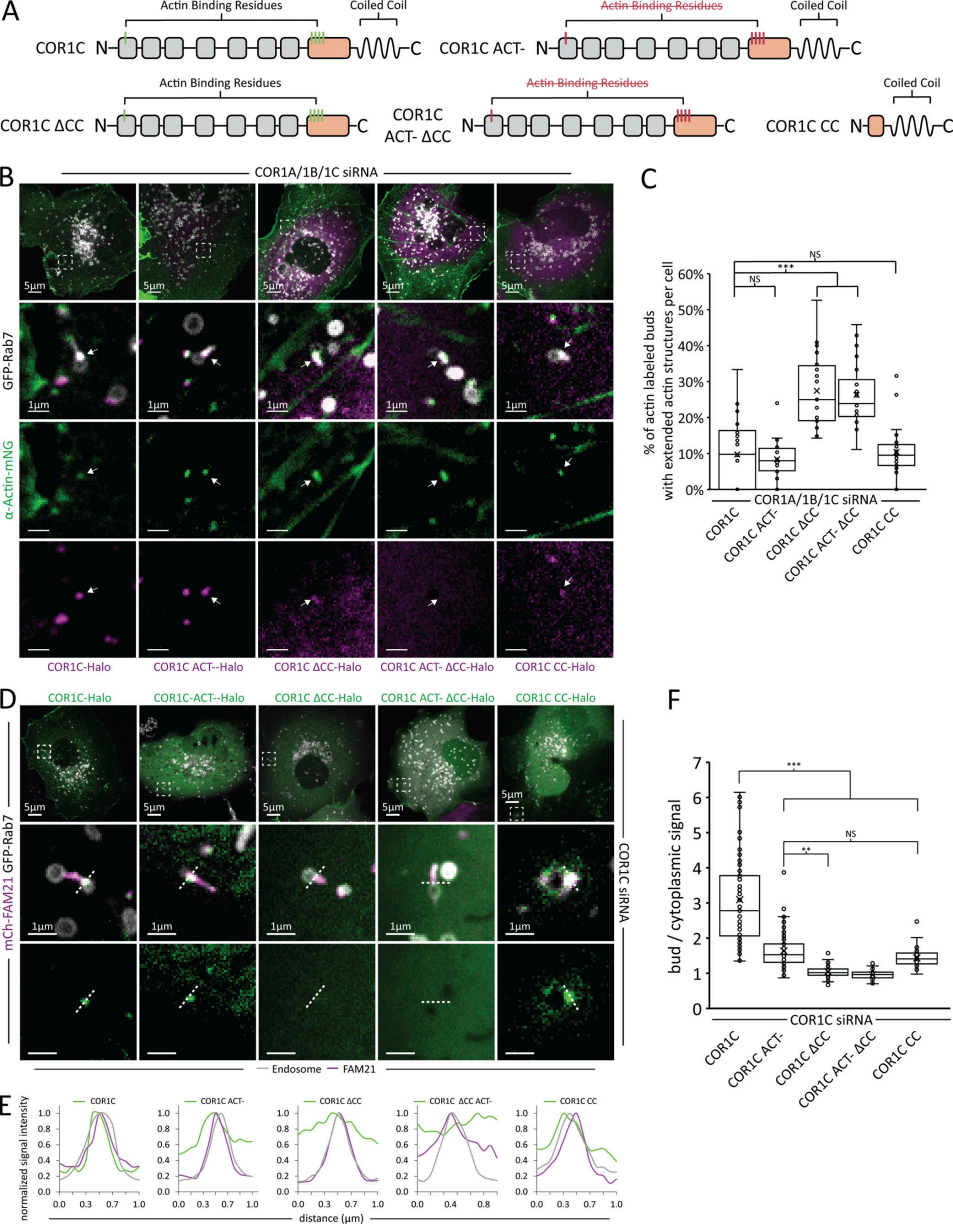
**The COR1C CC is necessary and sufficient for COR1C recruitment. (A)** Domain diagrams of the mutations made to test domain functionality in COR1C. ACT– indicates point mutations in actin-binding residues (R28D, K418E/K419E, K427E/K428E). ΔCC indicates a truncation removing the CC (COR1C residues 1–444). CC indicates predicted CC along with 30 upstream AAs (COR1C residues 414–474). **(B)** Representative images of COS-7 cells cotransfected with COR1A/1B/1C siRNA to deplete all type I coronins and with mCh-Rab7 (LE, gray), α actin-mNG (green), and either siRES COR1C-Halo, siRES COR1C ACT–-Halo, siRES COR1C ΔCC-Halo, siRES COR1C ACT– ΔCC-Halo, or siRES COR1C CC-Halo (magenta) to identify which domains are required to clear the extended actin structure from the distal bud. Magnified insets (5 × 5 μm) show representative examples of actin-positive endosome buds (at arrow). **(C)** Quantification of data in B. Graph shows percentage of actin-labeled buds with extended actin structures per cell from siRES COR1C-Halo: 480 endosomes in *n* = 22 cells; siRES COR1C ACT–-Halo: 578 endosomes in *n* = 21 cells; siRES COR1C ΔCC-Halo: 575 endosomes in *n* = 24 cells; siRES COR1C ACT– ΔCC-Halo: 549 endosomes in *n* = 22 cell; and siRES COR1C CC-Halo: 616 endosomes in *n* = 23 cells, performed in triplicate. **(D)** Representative images of COS-7 cells cotransfected with COR1C siRNA (for depletion), GFP-Rab7 (LE, gray), mCh-FAM21 (WASH complex, magenta), and siRES COR1C-Halo, siRES COR1C ACT–-Halo, siRES COR1C ΔCC-Halo, siRES COR1C ACT– ΔCC-Halo, or siRES COR1C CC-Halo (green) to measure the relative levels of recruitment to FAM21 marked buds for different COR1C mutants. Magnified insets (5 × 5 μm) of representative endosomes with FAM21 marked buds shown on right. Dashed line indicates where line scan analysis in D was done. **(E)** Line scan analysis of dashed lines shown in D are positioned to cross perpendicular to bud neck. Matching COR1C peaks indicate enrichment at the FAM21-labeled bud. Note that constructs lacking the CC do not form clear peaks. **(F)** Graph of data from experiment D; Halo signal enrichment at FAM21 buds relative to background is scored for the following samples: siRES COR1C-Halo: *n* = 79 endosomes in nine cells; siRES COR1C ACT–-Halo: *n* = 87 endosomes in nine cells; siRES COR1C ΔCC-Halo: *n* = 80 endosomes in nine cells; siRES COR1C ACT– ΔCC-Halo: *n* = 75 endosomes in 10 cells; and siRES COR1C CC-Halo: *n* = 67 endosomes in 11 cells, performed in triplicate. Note that a value of 1 indicates no enrichment over cytoplasmic background as in CC deletion. X indicates mean, and line indicates median. Statistical analyses were performed with one-way ANOVA, P value from Tukey’s test: *, P < 0.05; **, P < 0.01; ***, P < 0.001. Scale bars for whole cell = 5 μm; insets = 1 μm.

Although COR1C depletion was not sufficient to cause actin extension along the LE bud, we know that COR1C enriches at the bud neck of LEs (Fig. 1 J) and to a greater degree than COR1A, suggesting that COR1C might function specifically at the LE (Hoyer et al., 2018). We asked whether the reintroduction of COR1C would be sufficient to restrict actin to the base of the LE bud in type I coronin–depleted cells. COS-7 cells were cotransfected with COR1A/1B/1C siRNA (to deplete all three type I coronins), GFP-Rab7 (LEs), mCh-FAM21 (WASH buds), and an siRNA resistant Halo-tagged COR1C reexpression construct (siRES COR1C-Halo). Immunoblot analysis confirmed that COR1A/1B/1C were efficiently depleted and that the rescue construct was expressed at similar levels to endogenous COR1C (Fig. S2 A). We imaged live cells and calculated the mean percentage of actin-labeled buds with extended actin per cell (Fig. 3, B and C). The COR1C reexpression construct localized to the base of the bud and reduced actin extension down to levels not significantly different from control siRNA treatment (2.3% in control, Fig. 2 D, vs. 9.7% in WT, Fig. 3 C).

Next, we generated several Halo-tagged siRNA-resistant deletion mutants of COR1C to test which of its structural domains are required for actin disassembly and/or bud localization. Type I coronins contain several characteristic structural domains: a seven-bladed β propeller region formed from WD40 repeat domains, a unique domain, and a carboxy terminal CC (see cartoon of domain structure in Figs. 2 A and 3 A). The β propeller contains a single actin-binding domain, which is conserved among type I coronins. COR1C also has a nonconserved secondary actin binding site in its unique domain (Chan et al., 2012). The CC was shown to be uniquely essential for COR1C’s association with actin filaments (Spoerl et al., 2002). This CC was of particular interest because the single yeast coronin (Crn1), which also has two actin binding domains, uses the CC to bind and regulate ARP2/3 (Humphries et al., 2002). Mutants generated were as follows: an actin-binding deficient mutant (COR1C ACT–; R28D, K418E/K419E, K427E/K428E; these mutations are sufficient to abrogate actin binding in vitro; Chan et al., 2012), a C-term CC deletion mutant (COR1C ΔCC; residues 1–444 predicted to abrogate ARP2/3 binding based on homology to Crn1), a combination of the two (COR1C ACT– ΔCC), and a truncation containing only C-term CC (COR1C-CC; residues 414–474; Fig. 3 A). We cotransfected COS-7 cells with COR1A/1B/1C siRNAs to deplete all type I coronins, α-actin-mNG, and mCh-Rab7 (LE) and compared phenotypes upon reexpression of siRES full-length COR1C-Halo, siRES COR1C ACT–-Halo, siRES COR1C ΔCC-Halo, siRES COR1C ACT– ΔCC-Halo, or siRES COR1C CC-Halo (Fig. S2 and Fig. 3, B and C). Immunoblot analysis confirmed that endogenous COR1A/1B/1C were efficiently depleted and that the rescue constructs were expressed at similar levels to each other and to endogenous COR1C (Fig. S2, B and C). Cells were imaged live, and the mean percentage of actin-labeled buds with extended actin per cell was scored for each rescue condition (Fig. 3 C). Interestingly, mutants lacking the CC did not restore actin confinement to the bud neck to levels comparable with the WT control (Fig. 3 C, 9.7% for WT vs. 27% for COR1C ΔCC-Halo and 26% COR1C ACT– ΔCC-Halo). Strikingly, the CC alone was sufficient to reduce the extended actin to the same degree as WT (Fig. 3 C, 10 and 9.7%, respectively).

### The COR1C CC is necessary and sufficient for COR1C enrichment at LE buds

We measured to what extent COR1C mutants that rescue actin clearance from the distal bud are also able to enrich to the LE bud in the absence of extended actin structures. This analysis was performed in COR1C-depleted cells to avoid any chance of homodimerization with endogenous COR1C (Chan et al., 2012). COS-7 cells were cotransfected with COR1C siRNAs, GFP-Rab7 (LEs), mCh-FAM21 (WASH complex), and either siRES COR1C-Halo, siRES COR1C ACT–-Halo, siRES COR1C ΔCC-Halo, siRES COR1C ACT– ΔCC-Halo, or siRES COR1C CC-Halo. Cells were imaged live to visualize recruitment of the COR1C constructs to FAM21-labeled budding domains (Fig. 3 D). To score the enrichment of each mutant at FAM21 domains, we traced a region of interest (ROI) around the FAM21 signal on the bud and measured the fluorescence intensity of COR1C signal within the ROI (Fig. 3 F). We calculated the fold enrichment of COR1C signal at the ROI compared with its signal in the cytoplasm. On average, siRES full-length COR1C-Halo enriches threefold over background cytoplasmic signal, while deleting the CC domain completely disrupted recruitment of COR1C to the endosome bud (Fig. 3, E and F). By comparison, constructs with the actin-binding domain mutated were still recruited, but at a reduced level (1.5-fold over cytoplasmic background). Interestingly, the COR1C CC domain alone was still significantly recruited to the endosome bud, albeit at reduced levels compared with WT (Fig. 3, E and F). These data demonstrate that while the actin binding residues aid in recruitment to the FAM21-labeled endosome bud, they are not required. The C-terminal CC, however, is clearly both necessary and sufficient for COR1C enrichment on the LE bud. This corresponds well with its ability to rescue actin clearance, suggesting that these mutants are acting directly on endosomal actin (Fig. 2, B–D).

### COR1C regulates ARP2/3 complex activity at the endosome bud

**Figure 4.**
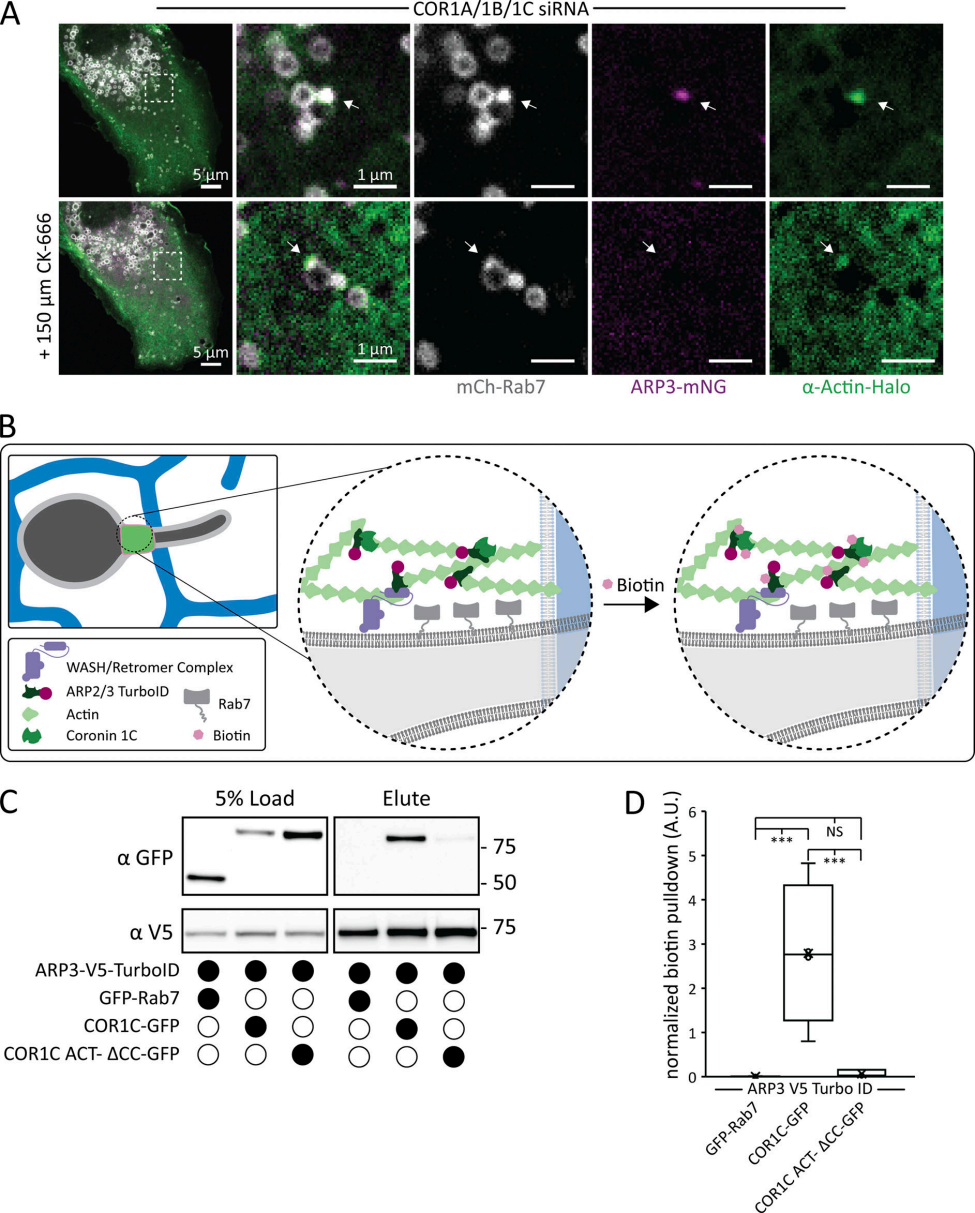
**COR1C regulates ARP2/3 complex activity at the endosome bud. (A)** Representative images of COS-7 cells cotransfected with COR1A/1B/1C siRNAs to deplete all type I coronins and with mCh-Rab7 (LE, gray), α actin-Halo (green), and ARP3-mNG (magenta). Buds with extended actin (at arrow) were tracked before and after addition of an ARP2/3 complex inhibitor (150 μM CK-666) in merged magnified insets on right (5 × 5 μm). *n* = 10 cells. **(B)** Diagram of the ARP3-V5-TurboID biotinylation experiment. HeLa cells were cotransfected with ARP2/3-V5-TurboID and GFP-Rab7, COR1C-GFP, or COR1C-ACT– ΔCC-GFP and treated with biotin, and then biotinylated proteins were bound and eluted from α-biotin beads. **(C)** Representative V5 and GFP immunoblot from experiment in B shows high levels of biotinylation for full-length COR1C compared with COR1C mutant or Rab7 control. **(D)** Quantification of immunoblot, as shown in C. Pulldown numbers were calculated by normalizing elute signal with the load signal, performed in triplicate. Statistical analyses were performed with one-way ANOVA, P value from Tukey’s test: ***, P < 0.001. Scale bars for whole cell = 5 μm; insets = 1 μm. Source data are available for this figure: SourceData F4.

Having established COR1C’s importance in confining actin, we probed by what mechanism COR1C regulates actin dynamics on the endosome bud before fission. The recruitment of COR1B to the ARP2/3 complex inhibits branched actin nucleation at the cell cortex (Cai et al., 2008; Cai et al., 2007). Thus, we hypothesized that COR1C might also disrupt branched actin on the endosome bud by binding and disrupting ARP2/3 activity. To test this, we asked whether the extended actin phenotype scored in type I coronin–depleted cells could be rescued by treatment with a drug that ectopically inhibits and displaces ARP2/3. We cotransfected COS-7 cells with COR1A/1B/1C siRNAs, ARP3-mNG (ARP2/3 complex), mCh-Rab7 (LE), and α-actin-Halo. We then imaged cells for 1 min at 2-s intervals to identify endosomes with extended actin structures. Next, we treated cells with 150 µM CK-666, an inhibitor that binds to ARP2/3 complex, locking it in an inactive state, and imaged 30 s after drug treatment (Hetrick et al., 2013). Inhibition of the ARP2/3 complex quickly depleted the actin and ARP3 marker signal on endosome buds (Fig. 4 A). These data demonstrate that limiting ARP2/3 complex activity is sufficient to reduce the extension of actin structures on endosome buds in type I coronin–depleted cells.

Having established the importance of ARP2/3 complex activity for the extended actin phenotype, we hypothesized that COR1C CC is essential for COR1C and ARP2/3 complex interaction given that the CC is both necessary and sufficient for localization and actin confinement. To test this hypothesis, we used a proximity labeling system, which makes use of the promiscuous biotin ligase TurboID (Branon et al., 2017). This system is well suited for capturing dynamic interactions between components on the quickly cycling actin structures present on the bud (Puthenveedu et al., 2010; Hoyer et al., 2018). We created a fusion protein between ARP2/3 complex subunit ARP3 and TurboID. We cotransfected HeLa cells with ARP3-V5-TurboID and COR1C-GFP, COR1C ACT– ΔCC-GFP, or a background endosomal marker, control GFP-Rab7 (Fig. 4 B). We treated transfected cells with biotin for 3 h, lysed the cells, and used biotin antibody agarose beads to enrich for biotinylated proteins. We eluted proteins from the beads and measured the amount of biotinylated protein in eluate and load fractions on anti-GFP immunoblots (Fig. 4 C). ARP3-V5-TurboID samples biotinylated COR1C-GFP at 40× the levels of the COR1C ΔCC-GFP mutant or the GFP-Rab7–negative control (Fig. 4, C and D).

**Figure S3.**
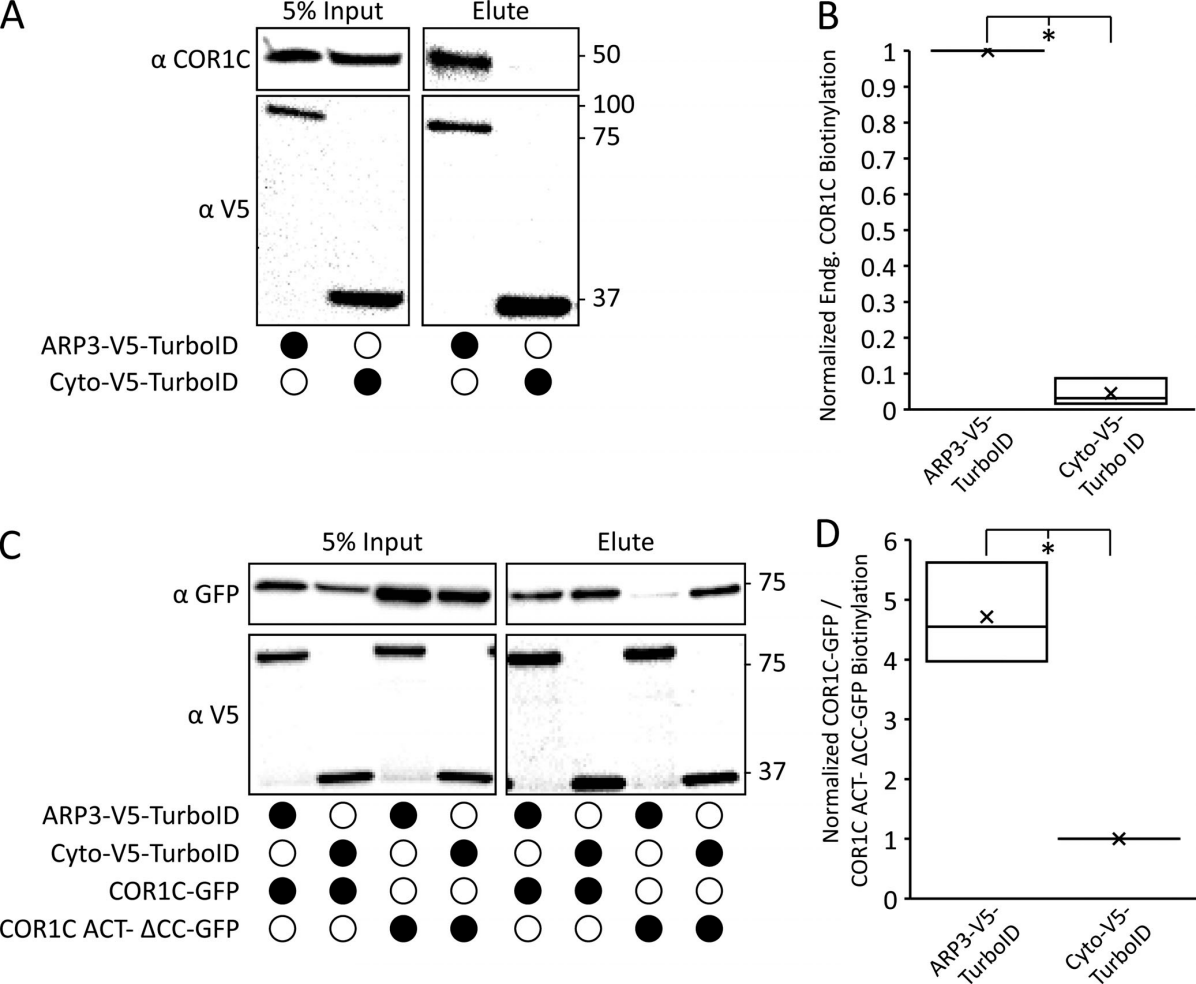
**Additional TurboID controls indicating that ARP3 TurboID activity is specific. (A)** Representative immunoblot showing that ARP3 V5 TurboID and a cytoplasmic nonspecific TurboID are able to biotinylate endogenous COR1C. Blots show the same samples run twice to blot for either endogenous COR1C or V5. Performed in triplicate. **(B)** Quantification of blots in A showing the amount of COR1C signal in the elute normalized first to the relevant TurboID V5 signal in the input and then to the max value within each replicate. **(C)** Representative immunoblot showing that ARP3 V5 TurboID has uniquely specific biotinylation of COR1C as shown by its ability to biotinylate only COR1C-GFP and not COR1C ACT– ΔCC-GFP. In contrast, the Cyto V5 TurboID biotinylates proteins nonspecifically in the cytoplasm as shown by similar biotinylation profiles for both COR1C-GFP and COR1C ACT–-ΔCC-GFP. Performed in triplicate. **(D)** Quantification of blots in C, from three replicates. Graph shows the ratio of COR1C-GFP to COR1C ACT–ΔCC-GFP signal in the elution. Signals in ratio were normalized to both relevant GFP and V5 input signals and then to the minimum value in each replicate. Statistical analysis was performed via two-tailed Student’s *t* test; *, P < 0.05. X indicates mean, and line indicates median. Source data are available for this figure: SourceData FS3.

To demonstrate that the biotinylation activity of ARP3-V5-TurboID was specific and could also biotinylate endogenous proteins, we also repeated the experiments as detailed above with a nonspecific construct localized to the cytoplasm, Cyto-V5-TurboID. We cotransfected HeLa cells with either ARP3-V5-TurboID or Cyto-V5-TurboID and either no additional exogenous bait (to probe for endogenous biotinylation) or addition of COR1C-GFP or COR1C ACT– ΔCC-GFP (Fig. S3). When we probed elution fractions for COR1C, we observed that only ARP3-V5-Turbo-ID was able to biotinylate endogenous COR1C (Fig. S3, A and B). The specificity of the biotinylation was further supported when we compared the degree to which COR1C-GFP versus COR1C ACT– ΔCC-GFP was biotinylated by ARP3-V5-TurboID or Cyto-V5-TurboID (Fig. S3, C and D). ARP3-V5-TurboID biotinylates COR1C-GFP to a much greater extent than COR1C ACT– ΔCC-GFP (Fig. S3, C and D; and Fig. 4, B and C). This is in contrast with the nonspecific activity of Cyto-V5-TurboID, which did not discriminate between COR1C-GFP and COR1C ACT– ΔCC-GFP constructs, because both are equally accessible in the cytoplasm (Fig. S3 C). These data suggest that the CC is required for ARP2/3 complex binding.

### The COR1C CC is necessary and sufficient to rescue LE fission and cargo sorting

**Figure S4.**
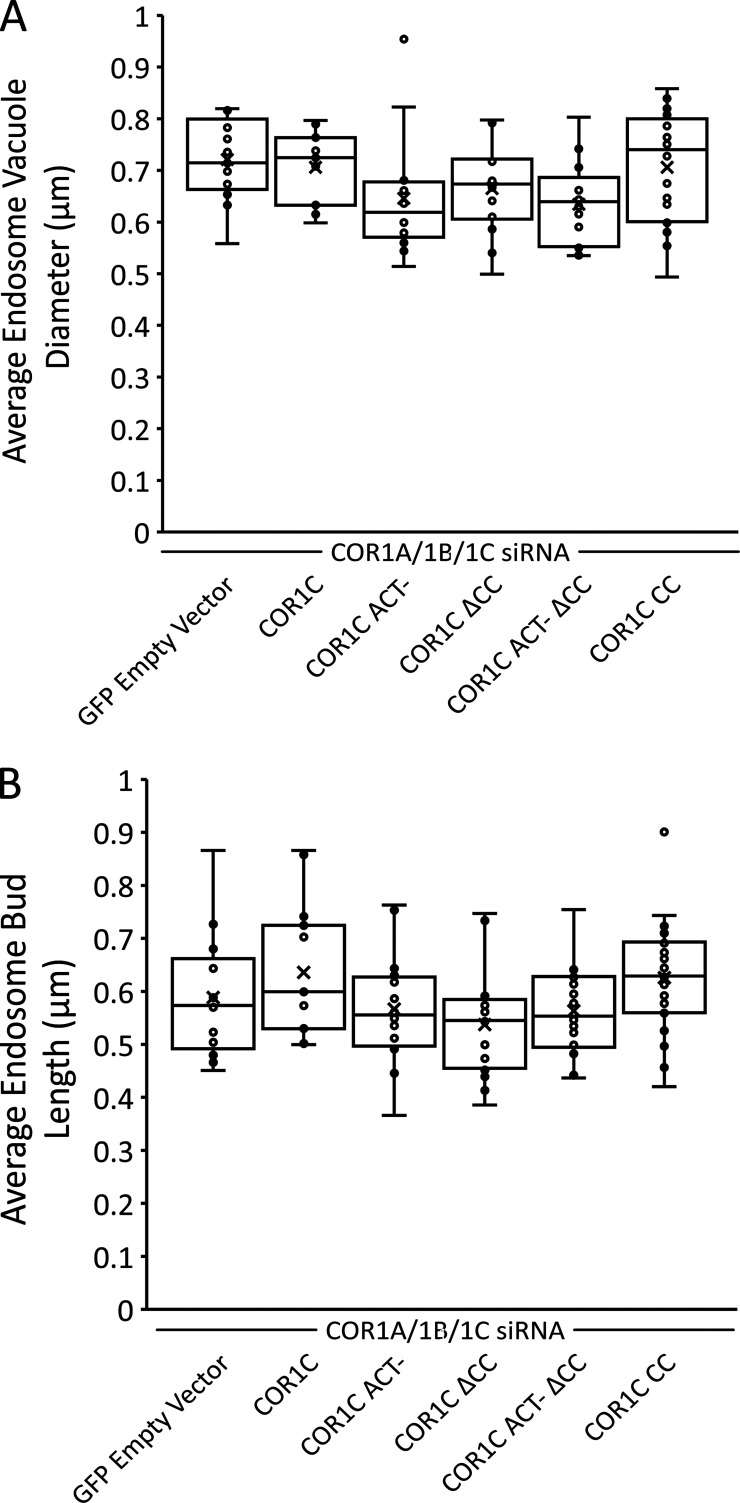
**Vacuole diameter and bud length are not changed significantly in KD rescue experiments. (A)** For each bud scored for fission in Fig. 5 C, vacuole diameter was also measured. Graph shows average vacuole diameter per cell, averaged per condition. No significant changes were observed in any condition, showing that only fission is affected and not other measures of endosome morphology. Data for graph from Halo E-vec: 139 endosomes in *n* = 15 cells; COR1C: 122 endosomes in *n* = 15 cells; COR1C ACT–: 213 endosomes in *n* = 17 cells; COR1C ΔCC: 281 endosomes in *n* = 18 cells; COR1C ACT– ΔCC: 296 endosomes *n* = 17 cells; and COR1C CC: 331 endosomes in *n* = 22 cells. X indicates mean, and line indicates median. Statistical analyses were performed with one-way ANOVA. **(B)** As in A, except FAM21-positive bud length was measured instead of vacuole diameter. Again, no significant changes were observed in any condition showing that only fission is affected and not other measures of endosome morphology. Data for graph from Halo E-vec: 139 endosomes in *n* = 15 cells; COR1C: 122 endosomes in *n* = 15 cells; COR1C ACT–: 213 endosomes in *n* = 17 cells; COR1C ΔCC: 281 endosomes in *n* = 18 cells; COR1C ACT– ΔCC: 296 endosomes *n* = 17 cells; and COR1C CC: 331 endosomes in *n* = 22 cells. X indicates mean, and line indicates median. Statistical analyses were performed with one-way ANOVA.

**Figure 5.**
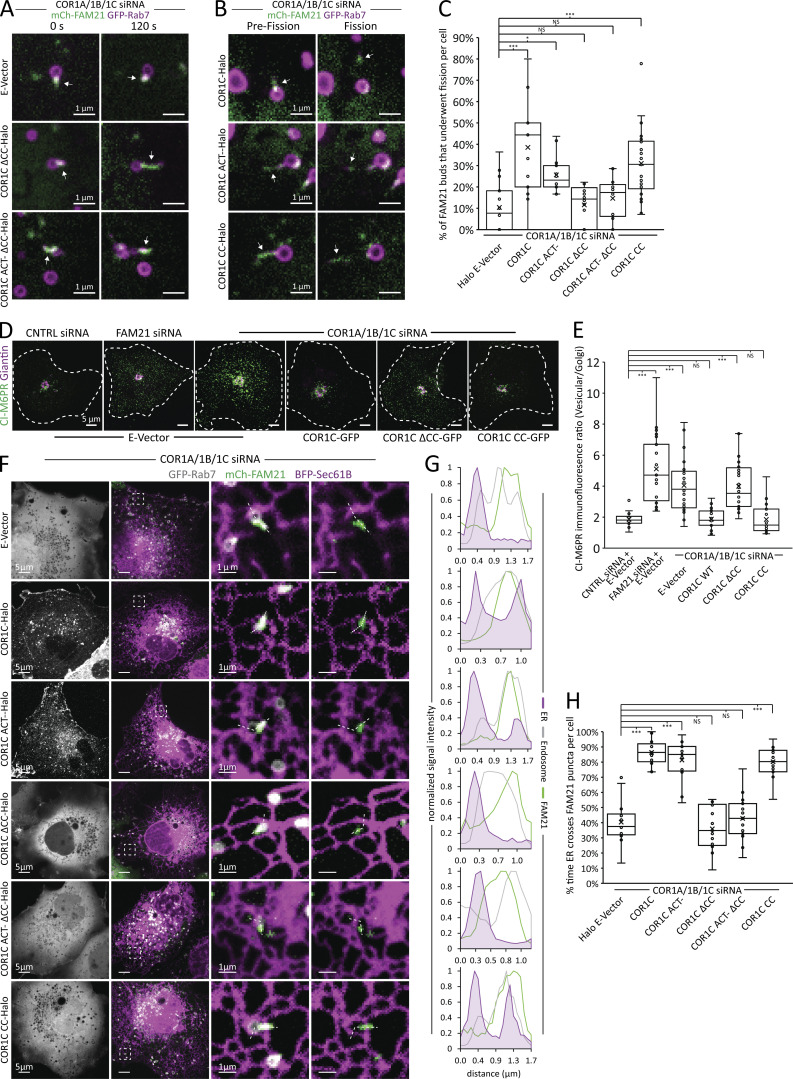
**The COR1C CC limits bud actin to facilitate ER contact, endosome fission, and CI-M6PR sorting. (A)** Representative images of LE buds stable for duration of acquisition in conditions that did not rescued fission rate (C). COS-7 cells were cotransfected with COR1A/1B/1C siRNAs to deplete all type I coronins and with GFP-Rab7 (LE, magenta), mCh-FAM21 (WASH complex, green), and with either Halo E-vec, siRES COR1C ΔCC-Halo, or siRES COR1C ACT– ΔCC-Halo. Arrows indicate bud of interest. **(B)** Representative images of LE fission events in conditions that rescued fission rate (C). COS-7 cells were cotransfected with COR1A/1B/1C siRNAs to deplete all type I coronins and with GFP-Rab7 (LE, magenta), mCh-FAM21 (WASH complex, green), and with either siRES COR1C-Halo, siRES COR1C ACT–-Halo, or siRES COR1C CC-Halo. Arrows indicate bud of interest. **(C)** Quantification of data in A and B. Graph shows percentage of FAM21-labeled LE buds that underwent fission per cell during a 2-min time lapse. Note that only constructs containing the CC were able to restore fission. Data for graph from Halo E-vec: 139 endosomes in *n* = 15 cells; siRES COR1C: 122 endosomes in *n* = 15 cells; siRES COR1C ACT–: 213 endosomes in *n* = 17 cells; siRES COR1C ΔCC: 281 endosomes in *n* = 18 cells; siRES COR1C ACT– ΔCC: 296 endosomes *n* = 17 cells; and siRES COR1C CC: 331 endosomes in *n* = 22 cells, performed in triplicate. **(D)** Representative images of the M6PR trafficking assay. The relative fluorescence intensity of internalized anti-CI-MPR antibody immunostaining reveals trafficking of internalized anti-CI-MPR to TGN in Cos7 cells cotransfected with control siRNA, COR1A/1B/1C siRNAs to deplete all type I coronins, or FAM21 siRNA and with either GFP E-vec, siRES COR1C-GFP, or siRES COR1C ΔCC-GFP (not depicted). Cells were stained to mark CI-M6PR (green) and Giantin (Golgi, magenta). Dispersed vesicular CI-M6PR signal is indicative of failure to recycle, whereas concentrated CI-M6PR signal at the Golgi indicates normal retrograde sorting. **(E)** Quantification of data in D. Graph shows the background-corrected ratio of CI-M6PR signal localized at the Golgi relative to the vesicular signal in the cytoplasm such that larger values indicate less efficient retrograde recycling. Data for graph from control siRNAs: *n* = 24 cells; FAM21 siRNAs: *n* = 23 cells; COR1A/1B/1C siRNAs + E-vec: *n* = 25; COR1A/1B/1C siRNAs + siRES COR1C: *n* = 24 cells; COR1A/1B/1C siRNAs + siRES COR1C ΔCC: *n* = 24 cells; COR1A/1B/1C siRNAs + siRES COR1C CC: *n* = 26 cells, performed in triplicate. **(F)** Representative images of COS-7 cells cotransfected with COR1A/1B/1C siRNAs to deplete all type I coronins and with GFP-Rab7 (LE, gray), mCh-FAM21 (WASH complex, green), BFP-Sec61β (ER, magenta), and either Halo E-vec, siRES COR1C-Halo, siRES COR1C ACT–-Halo, siRES COR1C ΔCC-Halo, siRES COR1C ACT– ΔCC-Halo, or siRES COR1C CC-Halo (gray, left panel). Magnified inset (5 × 5 μm) on right show representative examples of ER contact with endosomes. Note: Vacuolar contact with ER is always preserved, whereas ER contact with the FAM21 labeled bud is not. **(G)** Line scan analysis of dashed lines shown in D are positioned from the rear vacuolar contact across the length of the FAM21-labeled buds. Double ER peaks (shaded purple) are observed when bud contact is rescued. The first peak is always present and corresponds to the vacuolar contact. The second ER peak which aligns with the FAM21 indicates proper ER recruitment to the bud. Note that ER contact with bud is dependent on the presence of the CC domain. **(H)** Quantification of data in D. All FAM21-positive LE buds in areas with resolvable ER were tracked, and ER contact was scored as the percentage of time during a 2-min video that contact is maintained. Data for graph from Halo E-vec: 109 endosomes in *n* = 14 cells; COR1C: 134 endosomes in *n* = 17 cells; COR1C ACT–: 135 endosomes in *n* = 17 cells; COR1C ΔCC: 123 endosomes *n* = 17 cells; COR1C ACT– ΔCC: 105 endosomes in *n* = 15 cells; or COR1C CC: 137 endosomes in *n* = 19 cells, performed in triplicate. X indicates mean, and line indicates median. Statistical analyses were performed with one-way ANOVA, P value from Tukey’s test: *, P < 0.05; ***, P < 0.001. Scale bars for whole cell = 5 μm; insets = 1 μm.

**Figure S5.**
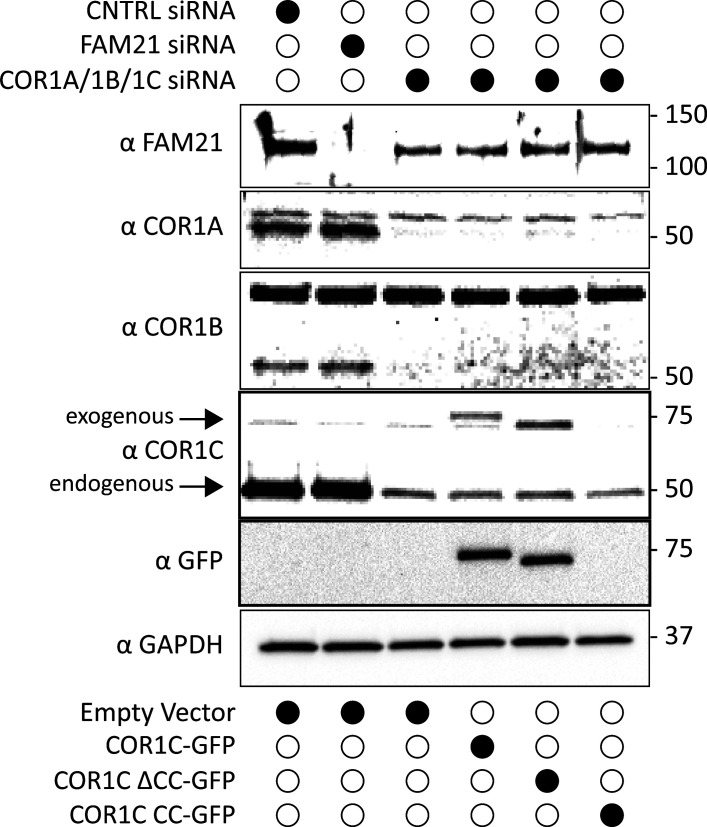
**Immunoblot of FAM21 depletion and type I coronin depletion and rescue in COS-7 cells for CI-M6PR sorting.** Immunoblots probed with antibodies indicated show efficient depletion of COR1A/1B/1C or FAM21 for experiments performed in Fig. 5, D and E. Blots were also probed for GFP to show exogenous rescue expression and GAPDH as loading control. Source data are available for this figure: SourceData FS5.

We showed that actin extension into the distal bud does not disrupt the assembly of cargo-sorting machinery components (Fig. 2 E). We hypothesized that although these components are present, actin must be confined to the bud neck for LE bud fission to occur. To test this hypothesis directly, we cotransfected COS-7 cells with COR1A/1B/1C siRNAs to deplete all type I coronins, GFP-Rab7 (LE), mCh-FAM21 (WASH complex), and either a Halo empty vector (E-vec) control, siRES COR1C-Halo, siRES COR1C ACT–-Halo, siRES COR1C ΔCC-Halo, siRES COR1C ACT– ΔCC-Halo, or siRES COR1C CC-Halo. We collected 2-min time-lapse videos at 2-s intervals, and each FAM21-positive bud was scored for length, vacuole diameter, and fission (Figs. S4 and 5 C). There were no significant changes to either bud length or vacuole diameter (Fig. S4). Rescue with an E-vec showed that type I coronin depletion results in a threefold reduction in fission rate (10%; Fig. 5, A and C; Hoyer et al., 2018). Reintroduction of full-length COR1C was sufficient to rescue fission rate (39%; Fig. 5, B and C). The mutants that lack the CC domain did not restore fission rate above the E-vec control, resulting in FAM21-labeled buds that were stable for the duration of the time-lapse (Fig. 5 A): COR1C ΔCC, COR1C ACT– ΔCC-Halo (12 and 15% vs. 11%, respectively; Fig. 5 C). The COR1C CC proved both necessary and sufficient to rescue the FAM21-positive bud fission rate to levels comparable with WT rescue (Fig. 5, A and C).

Having established the importance of the COR1C CC for FAM21-marked bud fission, we hypothesized that retrograde membrane cargo flow would be limited upon depletion of type I coronins. To test this, we measured recycling of the cation-independent mannose 6 phosphate receptor (CI-M6PR), which relies on the WASH complex for retrograde transport to the Golgi (Dong et al., 2016; Duleh and Welch, 2010). We cotransfected COS-7 cells with COR1A/1B/1C siRNAs, FAM21 siRNAs (control), or control siRNAs and GFP E-vec (control; Fig. S5). These cells were then treated with CI-M6PR antibody for 1 h before they were rinsed to remove unbound antibody and fixed. Using immunofluorescence, we probed the intracellular distribution of CI-M6PR signal relative to the Golgi in these fixed cells (Fig. 5 D). We observed that in both the FAM21 and type I coronin depletions, CI-M6PR signal failed to accumulate at the Golgi as in the control siRNA–treated cells, instead remaining trapped in vesicles distributed broadly across the cytoplasm (Fig. 5, D and E). We hypothesized that as with fission, the COR1C CC would be both necessary and sufficient to rescue retrograde sorting of CI-M6PR. To test this, we cotransfected COS-7 cells with COR1A/1B/1C siRNAs, and with either siRES COR1C-GFP, siRES COR1C ΔCC-GFP, or siRES COR1C CC-GFP. As before, cells were treated with CI-M6PR antibody for an hour, fixed, and stained to measure retrograde recycling efficiency (Fig. 5 D). Reintroduction of either COR1C-GFP or COR1C CC-GFP was sufficient to restore retrograde recycling to a degree similar to that of control siRNA–treated cells (Fig. 5 E). In contrast, reintroduction of COR1C ΔCC-GFP was unable to rescue retrograde sorting of CI-M6PR (Fig. 5, D and E). As with fission, the COR1C CC proved both necessary and sufficient to restore efficient retrograde recycling of CI-M6PR.

### The COR1C CC limits bud actin to facilitate ER contact

We have shown that type I coronin depletion results in extended actin on the buds, a reduction in endosome fission rate, and reduced retrograde recycling of the CI-M6PR. Endosome fission at WASH complex marked buds is regulated by MCSs with ER tubules (Rowland et al., 2014; Hoyer et al., 2018; Allison et al., 2013, 2017; Dong et al., 2016). Thus, we hypothesized that type I coronin depletion and actin extension might impede the formation of ER-endosome MCSs between ER tubules and the endosome bud. To test this, we imaged the ER and endosomes in live cells depleted of all type I coronins. We cotransfected COS-7 cells with COR1A/1B/1C siRNAs, BFP-Sec61B (ER marker), GFP-Rab7 (LEs), mCh-FAM21 (WASH complex), and with either a Halo E-vec (control) or siRNA-resistant COR1C-Halo (Fig. 5 F). We imaged cells for 2 min at 2-s intervals. We scored the percentage of frames (during the 2-min time-lapse) that the ER tubules tracked with the FAM21-positive endosome buds. This allowed us to calculate a percentage contact per endosome bud, which was averaged per cell (Fig. 5 H). The data revealed a clear impairment of ER contact with the FAM21 marked endosome buds in the E-vec control (40%) compared with the WT rescue (86%; Fig. 5 H). This supports the idea that the extended actin structures on endosome buds hinder proper contact site formation. Interestingly, the vacuole ER MCS remained largely unchanged, as can be seen in the line scans along the vacuole and bud, where the first ER peak corresponds to the vacuole MCS (Fig. 5 G). This suggests that type I coronin depletion does not generally inhibit ER-endosome MCS formation, but specifically compromises the bud MCS.

**Figure 6.**
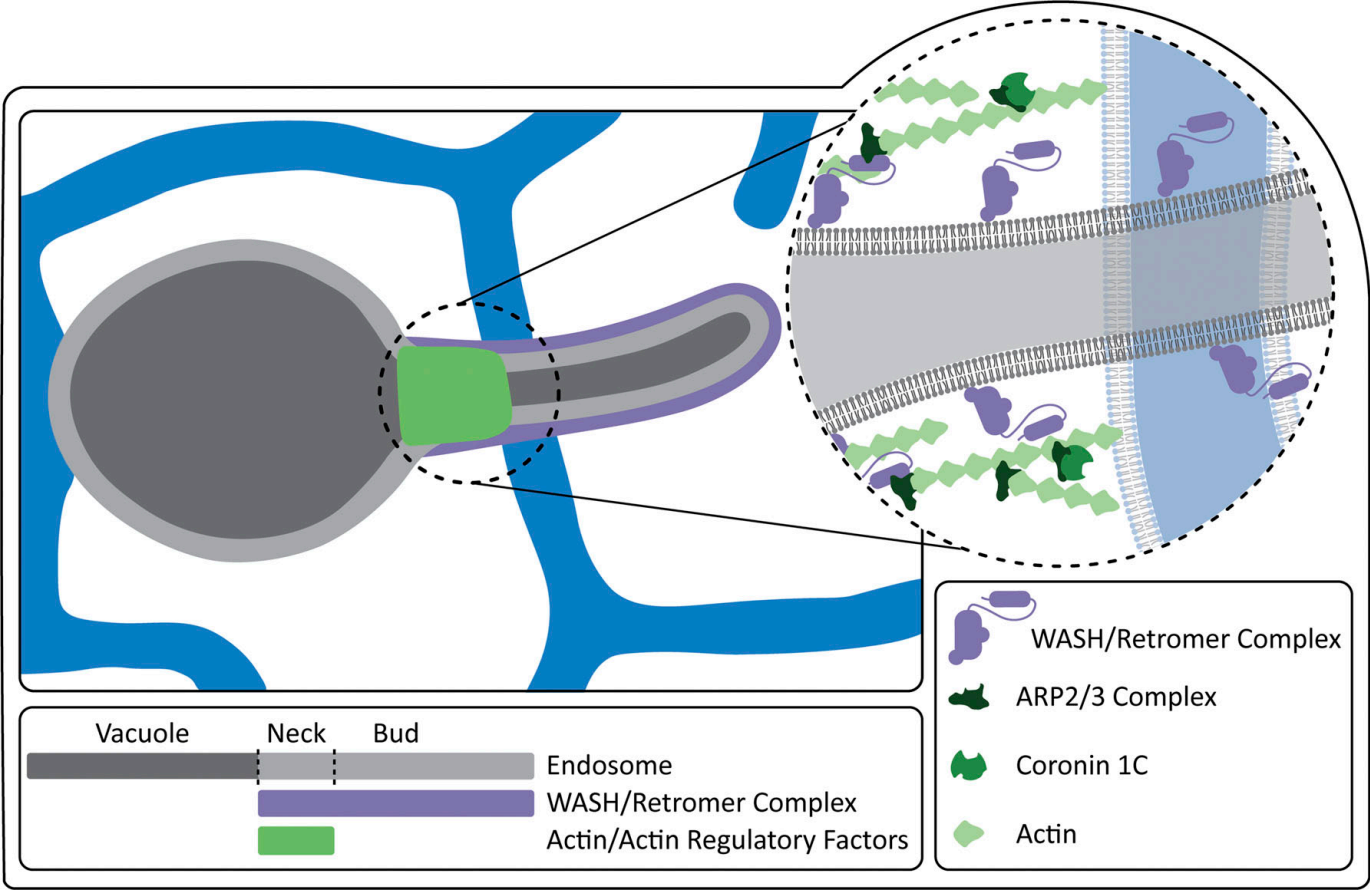
**Model of actin/actin regulatory factors position and ER MCS just before fission.** The WASH complex recruits and activates ARP2/3 to form a stabilizing actin structure on the endosome bud. COR1C is recruited to these actin structures to bind and counter act ARP2/3 actin nucleation via its CC. This results in an actin structure large enough to stabilize the bud, allowing cargo to sort, but small enough to allow ER recruitment and bud fission.

Because the COR1C CC domain is necessary and sufficient to rescue actin clearance from the distal bud and LE fission, we investigated if it could also rescue ER contact with FAM21-labeled buds. As before, we cotransfected COS-7 cells with COR1A/1B/1C siRNAs to deplete all three type I coronins, BFP-Sec61B (ER), GFP-Rab7 (LE), and mCh-FAM21 (WASH complex), and reintroduced either siRES COR1C ACT–-Halo, siRES COR1C ΔCC-Halo, siRES COR1C ACT– ΔCC-Halo, or siRES COR1C CC-Halo. We scored ER contacts with FAM21-positive endosome buds in 2-min time-lapse videos as before (Fig. 5 F). The COR1C CC proved to be both necessary and sufficient to rescue ER tubule contact with the FAM21-labeled endosome bud. In cells rescued with mutants lacking the CC, ER contact was not restored to levels significantly different from the E-vec control (Fig. 5 H; E-vector 40%, ΔCC 36%, and ACT– ΔCC 43%). Cells rescued with the COR1C ACT– or COR1C CC constructs showed restored ER-LE bud MCSs (81 and 80%, respectively) to the same degree as WT rescue (86%; Fig. 5 H). Taken together, these results support the model where the COR1C CC domain functions to restrict ARP2/3-mediated actin extension along the endosome bud to allow ER MCS formation during ER-associated endosome fission (Fig. 6).

## Discussion

Early studies indicated that branched actin assembly on buds is required for efficient cargo recycling from the endosome and proposed it was required for fission based on actin function during endocytosis (Simonetti and Cullen, 2019). Puzzlingly, the presence of actin on an endosome bud was subsequently shown to increase bud stability in a manner seemingly opposed to fission (Puthenveedu et al., 2010). The new model proposed that actin-mediated bud stabilization delayed the fission process in order for slow-diffusing cargos to be sorted into the bud. Here, we examined actin and fission directly and discovered that actin and its regulators organize the position of fission by defining membrane availability for ER MCS formation. We used time-lapse microscopy to show that Arp2/3 nucleated branched actin is restricted to the bud neck in a mechanism that depends on type I coronins. When type I coronins are depleted from the cell, the endosomal actin structures extend along the entire length of the bud, preventing efficient ER-LE bud contact, thus stalling endosome fission and preventing the efficient recycling of retrograde cargo. Strikingly, we found that the CC domain of COR1C alone is sufficient to rescue all measured phenotypes and functions of type I coronins at the bud. By probing the functionality of the COR1C’s CC, we discovered that the CC is required for ARP2/3 interaction. Further, we showed that by ectopically and acutely inhibiting the ARP2/3 complex, we could reverse the effects of type I coronin depletion on bud actin structure. Together, these data support a model whereby COR1C CC binds and regulates the activity of ARP2/3 complex at the endosome bud and suggest that confinement of this actin-ARP2/3-COR1C structure to the bud neck is essential for bud fission.

The unique enrichment of COR1C at the endosome bud and its capacity to rescue actin confinement and fission defects in a type I coronin depletion suggests that COR1C has a specialized function in regulating endosomal actin. Although our data do indicate that COR1A and COR1B can prevent the more dramatic actin polymerization phenotypes, we previously showed that COR1C depletion alone is sufficient to reduce endosome fission rate by a mechanism that remained unexplored until now (Hoyer et al., 2018). This, combined with the diversity of mammalian coronins, points to the possibility that other specialized branched actin structures in the cell might also have a designated coronin regulator (Chan et al., 2011).

Given that the actin structure is thought to stabilize the endosome bud, we were initially surprised that actin and its associated regulatory factors are not completely cleared from the endosome bud during fission. Rather, they are maintained in a stable but restricted location at the neck of the endosome bud. This suggests that the size of the actin structure is key to the slow receptor sorting process (Puthenveedu et al., 2010). The actin structure must be present to stabilize the bud and slow fission, but it must also be confined so that the buds remain fission competent. Actin, ARP3, and COR1C rarely leave with a departing bud remaining on the vacuolar half of the fission event. This is unlike the WASH complex (FAM21), which labels the entire bud and always marks the departing bud. If the membrane occupied by the actin structure were fission competent, we would expect to see actin signal departing with the bud at a much higher rate. This partitioning suggests that the actin structure defines where fission can occur: specifically, at a distal position on the bud that is not occupied by actin.

The stability of the actin/ARP2/3/COR1C domain is also intriguing because it indicates that endosomes maintain a sorting domain that can perform multiple rounds of cargo sorting, bud formation, and fission. This domain’s stability and partitioning parallels observations made about components of ER exit sites, specifically, that COPII collars remain stably associated with the ER rather than leaving with the departing Golgi-bound vesicle (Westrate et al., 2020; Mccaughey et al., 2019; Carter et al., 2020). This presents an interesting paradigm in which trafficking domains are maintained stably on origin membranes, and cargos are concentrated at these points rather than the reverse. It will be interesting to explore if multiple rounds of bud formation can occur from a single stable actin/ARP2/3/COR1C domain.

ER regulation of endosomal lipid composition is well established and is linked with a variety of downstream changes on the endosome (Wilhelm et al., 2017; Alpy et al., 2013; Rocha et al., 2009; Eden et al., 2010; Dong et al., 2016). Interestingly, lipid composition has been directly linked with regulation of actin polymerization on cellular membranes including endosomes (Hong et al., 2015; Di Paolo and De Camilli, 2006). This is most directly illustrated by work showing that an ER-endosome MCS forms by interactions between vesicle-associated membrane protein-associated protein VAP-A and VAP-B and an endosomal cargo sorting component, SNX2. This MCS facilitates changes in phosphoinositide composition on the endosome membrane, resulting in decreased WASH activity and thus less actin proliferation (Dong et al., 2016). This might fit well with our data showing the clear difference in WASH complex and ARP3 enrichment along the bud. Perhaps the ER, once recruited to a forming bud, actively alters distal bud lipid composition to further limit WASH activation and F-actin extension. These data also suggest intriguing feedback loops in which ER contact both regulates and is regulated by endosomal actin.

Our data indicate COR1C has an antagonistic regulatory interaction with ARP2/3 once the branched actin structure has been formed. As we discussed earlier, it is possible that other factors are also limiting ARP2/3 recruitment and activation. We show that WASH complex subunit FAM21 localizes along the entire length of the bud, but ARP3 is confined to the bud neck. This contrast hints that some other factors limit where ARP2/3 can bind and be activated by WASH complex. This factor could work in concert with COR1C, limiting where branched structures can be initiated, while COR1C limits the extent of polymerization of already seeded actin structures. Such a protein would also be an excellent candidate as an endosome localized ER tether since our data strongly indicate that the ER is recruited to non–actin coated membrane to drive the fission process.

## Materials and methods

### Plasmids and reagents

GFP-Rab7 and mCh-Rab7 were described previously (Rowland et al., 2014). Human Rab7 was PCR amplified from human cDNA and cloned into AcGFP/mCherry-C1 with XhoI/HindIII sites. Halo-Rab7 was generated by first PCR amplifying the Halo tag and cloning into AcGFP-C1 via AgeI/SacI sites to generate AcHalo-C1. Primers were JS75 and JS76 (Table S1). Rab7 was PCR amplified from mCh-Rab7 and cloned into AcHalo-C1 via HindIII/KpnI sites. Primers used were JS73 and JS74 (Table S1). COR1C-GFP was gift from Dr. Manojkumar Puthenveedu and Dr. Mark von Zastrow (University of California, San Francisco, San Francisco, CA; Puthenveedu et al., 2010). SiRes COR1C-Halo was generated mutating COR1C-GFP by four rounds of site-directed mutagenesis to remove siRNA binding to generate siRES COR1C-GFP, which was subcloned into Halo-N1 via EcoRI/BamHI to generate siRES COR1C-Halo. SiRES COR1C ACT–-Halo was generated by four rounds of site directed mutagenesis of COR1C-GFP to generate COR1C ACT–-GFP. Primers used were JS29–JS32 and JS34–JS37 (Table S1). COR1C ACT–-GFP was then mutated by four rounds of site-directed mutagenesis to remove siRNA binding to generate siRES COR1C ACT–-GFP, which was subcloned into Halo-N1 via EcoRI/BamHI to generate siRES COR1C ACT–-Halo. Primers used were JS41–JS48 (Table S1). SiRES COR1C ΔCC-Halo was generated by PCR amplification of COR1C-GFP to generate COR1C ΔCC-GFP. Primers used were JS19 and JS108 (Table S1). COR1C ΔCC-GFP was then mutated by four rounds of site-directed mutagenesis to remove siRNA binding to generate siRES COR1C ΔCC-GFP, which was subcloned into Halo-N1 via EcoRI/BamHI to generate siRES COR1C ΔCC-Halo. Primers used were JS41–JS48 (Table S1). SiRES COR1C ACT– ΔCC-Halo was generated by four rounds of site-directed mutagenesis of COR1C ΔCC-GFP to generate COR1C ACT– ΔCC-GFP. Primers used were JS29–JS32 and JS34–JS37 (Table S1). COR1C ACT– ΔCC-GFP was then mutated by four rounds of site-directed mutagenesis to remove siRNA binding to generate siRES COR1C ACT– ΔCC-GFP, which was subcloned into Halo-N1 via EcoRI/BamHI to generate siRES COR1C ACT– ΔCC-Halo. Primers used were JS41–JS48 (Table S1). COR1C CC-Halo was generated by PCR amplified from COR1C-GFP and then cloned into Halo-N1 via HindIII/KpnI sites. Primers used were JS109 and JS110 (Table S1). mEmerald-ARP3-N-12 was a gift from Michael Davidson (MagLab, Tallahassee, FL; plasmid #53995; Addgene). mCh-FAM21 was described previously (Rowland et al., 2014). Briefly, it was subcloned from shFAM21/HA-YFP-FAM21 which came as a gift from Dr. Daniel Billadeau (Mayo Clinic, Rochester, MN). α-Actin-mNG and α-actin-Halo were generated by PCR amplification from actin Chromobody TagGFP (Chromotek, acg) and cloning into mNG-N1 or Halo-N1 via XhoI/KpnI sites. Primers used were JS69 and JS70 (Table S1). V5 TurboID-N1 was generated by PCR amplification from V5-TurboID-NES which was a gift from Alice Ting (Stanford University, Stanford, CA plasmid #107169; Addgene) and cloning into ARP3-mNG via AgeI/NotI sites. Primers used were JS106 and JS107 (Table S1). FLAG-ADRB2-mNG was generated by PCR amplification from pcDNA3 Flag β-2-adrenergic-receptor, which was a gift from Robert Lefkowitz (Duke University, Durham, NC plasmid #14697; Addgene) and cloning into mNG-N1 via HindIII/PstI sites. BFP-Sec61b was subcloned from AcGFP-Sec61b into mTagBFP-C1 using BglII/EcoRI sites (Zurek et al., 2011; Shibata et al., 2008). GFP-PS35 was a gift from Melissa Hoyer (Harvard Medical School, Boston, MA).

For immunoblotting, rabbit COR1C polyclonal antibody (14749-1-AP; Proteintech) was used at 1:2,000; rabbit COR1B polyclonal antibody (ab119714; Abcam) at 1:2,000; rabbit COR1A polyclonal antibody (ab123574; Abcam) at 1:2,000; muse V5 monoclonal antibody (R960CUS; Thermo Fisher Scientific) at 1:2,000; mouse GFP monoclonal antibody (Clontech Labs 3P Living Colors A.v. Monoclonal Antibody [JL-8], NC9777966; Thermo Fisher Scientific) at 1:2,000; rabbit FAM21C polyclonal antibody (ABT79, lot #3560681; Millipore) at 1:1,000; rabbit GAPDH antibody (G9545; MilliporeSigma) at 1:100,000; anti-rabbit IgG (whole molecule)-peroxidase antibody produced in goat (A6154; Sigma-Aldrich) at 1:6,000; and anti-mouse IgG (whole molecule)-peroxidase antibody produced in goat (A4416; Sigma-Aldrich) at 1:3,000. For immunofluorescence, mouse IGFR2 (CI-M6PR) monoclonal antibody (MA1-066; Invitrogen) was used at 1:1,000; rabbit Giantin antibody (PRB-114C; BioLegend) at 1:500; goat anti-rabbit IgG (H + L) Cross-Adsorbed Secondary Antibody, Alexa Fluor 405 (cat #A-31556; Thermo Fisher Scientific) at 1:300; and donkey anti-mouse IgG (H + L) Antibody, Alexa Fluor 594 Conjugated (cat #A-21203; Thermo Fisher Scientific) at 1:300.

### Transfection

For imaging, COS-7 cells (ATCC; tested for mycoplasma before delivery and freezing) were seeded on 35-mm glass-bottom dishes (D35-20-1.5-N; Cellvis) in DMEM (12430-054; GIBCO) containing 10% FBS, 50 U/ml penicillin, and 50 μg/ml streptomycin (15,070,063; Invitrogen) for 16 h and then transfected with plasmids using Lipofectamine 3000 following the manufacturer’s protocol. Briefly, two separate 250-μl mixes were prepared with Opti-MEM (31985-088; Invitrogen). One mix received plasmids intended for transfection and 2 μl P3000 reagent per μg of plasmid. The second mix received 5 μl Lipofectamine 3000. These mixes were combined after a 5-min room temperature incubation. This was followed by a 20-min room temperature incubation, whereupon the combined mix was added to the imaging dish dropwise. Before addition of transfection mix, cells were rinsed once with 1× PBS and then placed in 1.5 ml Opti-MEM. Cells were incubated with transfection mix for 5 h, rinsed once with 1× PBS, and placed in 2 ml DMEM. Cells were then imaged 16 h later in 1.5 ml FluoroBrite DMEM (cat #A18967-01; GIBCO) containing 10% FBS, 25 mM Hepes, and 1× GlutaMAX (35,050,061; GIBCO). Cells transfected with halo tag had 100 nM Janelia Fluor 646 halo ligand, provided by Luke Lavis (Janelia Research Facility, Ashburn, VA), added to the imaging medium and were incubated with ligand for 30 min before imaging.

For imaging, ADRB2 localization protocol was adapted from Puthenveedu et al. (2010). Firefly cells were transfected with FLAG-ADRB2-mNG as detailed above. 30 min before imaging, cells were treated with 10 μM isoproterenol to induced endocytosis of ADRB2.

The following concentrations were used for each plasmid: 25 ng/ml mCh-Rab7; 25 ng/ml GFP-Rab7 (imaging and TurboID); 25 ng/ml Halo-Rab7; 25 ng/ml COR1C-Halo; 50 ng/ml siRES COR1C-GFP (CI-M6PR Rescue); 50 ng/ml SiRES COR1C-Halo (Rescue); 50 ng/ml SiRES COR1C ACT–-Halo (Rescue); 75 ng/ml SiRES COR1C ΔCC-Halo (Rescue); 75 ng/ml siRES COR1C ΔCC-GFP (CI-M6PR Rescue); 75 ng/ml SiRES COR1C ACT–-ΔCC-Halo (Rescue); 50 ng/ml SiRES COR1C CC-Halo (Rescue); 125 or COR1C-GFP (TurboID); 125 ng/ml COR1C ACT–-ΔCC-GFP (TurboID); 125 ng/ml ARP3-V5-TurboID; 45 ng/ml Cyto-V5-TurboID; 75 ng/ml ARP3-mEm; 250 ng/ml mCh-FAM21; 150 ng/ml GFP VPS35; 50 ng/ml α-actin-mNG; 200 ng/ml FLAG-ADRB2-mNG; 50 ng/ml α-actin-Halo; and 225 ng/ml BFP Sec61β.

### Knockdown (KD) with siRNA

Cells were seeded in 2-ml wells, as described previously, for 16 h. Cells were then transfected with siRNA at concentrations listed below with DharmaFECT 1 transfection reagent in DMEM containing 10% FBS for 6 h. Cells were allowed to recover for 40 h before transfecting with the same amount of siRNA and plasmids marking structures of interest, as described previously. After transfection, cells were split into an imaging dish and 2 ml for KD confirmation via Western blot.

Cells were transfected with 25 nM negative control siRNA (single KD) or 75 nM negative control siRNA (type I coronin KD; AM4635; Ambion) or 25 nM siRNA against COR1C (ON-TARGETplus SMARTPool L-017331-00-0010; Dharmacon), 25 nM siRNA against COR1A (ON-TARGETplus SMARTPool L-012771-00-0010; Dharmacon), 25 nM siRNA against COR1B (ON-TARGETplus SMARTPool L-010493-01-0010; Dharmacon), and 25 nM siRNA against FAM21 (ON-TARGETplus SMARTPool L-029678-01-0005; Dharmacon).

### Microscopy

Cells were imaged via a spinning disk confocal microscope or confocal line scanning microscope. The spinning disk confocal microscope consists of a Nikon eclipse Ti2 inverted microscope body equipped with a PSF unit, a Yokagowa CSU-X1 spinning disk confocal scanner, an Andor iXon 897 electron-multiplying charge-coupled device 512 × 512 camera, and OBIS 405-, 488-, 561-, and 640-nm lasers. Images were acquired with a 100× 1.45-NA Plan Apo objective using Micro-Manager software and ImageJ (National Institutes of Health). For Fig. 1, D–I, an Airyscan LSM 880 was used; all other images were acquired on spinning disk detailed above. The line scanning confocal consists of Zeiss Axio Observer inverted fluorescence microscope body equipped with LSM 880 and Airyscan detector. Images were acquired with 63× 1.4-NA Plan Apo objective Zeiss Zen Software.

### Extended actin structures quantification

To count the number of extended actin structures on endosomes, COS-7 cells in different KDs or KD rescue treatments were transfected with either GFP-Rab7 mCh-Rab7 (LE) and α-actin-mNG (actin). Cells were imaged for 1 min at 2-s intervals. The number of actin-labeled buds in each cell over the time lapse was counted. The number of those buds that qualified as extended actin buds was also counted. Extended actin buds were defined as buds longer than 250 nm with actin enrichment that covered more than half the bud for more than three consecutive frames. The ratio of actin labeled buds to extended actin buds was calculated per cell.

### COR1C endosome enrichment quantification

To measure COR1C enrichment, COS-7 cells were transfected with GFP-Rab7, mCh-FAM21, and COR1C siRNA and rescued with SiRES COR1C-Halo, SiRES COR1C ACT–Halo, SiRES COR1C ΔCC-Halo, SiRES COR1C ACT–ΔCC-Halo, or SiRES COR1C CC-Halo. Cells were imaged for 1 min at 2-s intervals. For every resolvable FAM21-positive endosome bud, the FAM21 signal was traced, and raw integrated density was measured in the COR1C channel. Local cytoplasmic signal was collected via measuring raw integrated density of a 1-μm-diameter circle that was placed in a proximal cytoplasmic area without endosomes. Background signal was collected by measuring raw integrated density of a 1-μm-diameter circle outside the cell. Measurements were normalized by area, and then background signal was subtracted from the bud and cytoplasmic signals. Enrichment over cytoplasm was calculated by dividing background-corrected bud signal by background-corrected cytoplasmic signal.

### Endosome fission analysis

Endosome fission at FAM21-positive buds was quantified as in Hoyer et al. (2018). Briefly, COS-7 cells were transfected with GFP-Rab7, mCh-FAM21, COR1A/1B/1C siRNA, and rescued with either SiRES COR1C-Halo, SiRES COR1C ACT–-Halo, SiRES COR1C ΔCC-Halo, SiRES COR1C ACT–-ΔCC-Halo, or SiRES COR1C CC-Halo. Cells were imaged for 2 min at 2-s intervals. All FAM21-positive endosome buds in resolvable regions of the cell were tracked for the duration of the time-lapse. Only endosomes with a clear distinction between vacuole and bud were measured. For each, the bud length, vacuole diameter, and whether or not fission occurred were recorded. To qualify as a fission event, buds had to separate clearly from vacuole and be resolvable in the post-fission frame. The number of FAM21 positive fission events was divided by the total number of FAM21-positive buds to give a per-cell fission percentage.

### TurboID proximity labeling analysis

Proximity labeling was adapted from Wu and Voeltz (2021). HeLa cells (ATCC; CCL-2, tested for mycoplasma before delivery and freezing) were transfected with ARP3-V5-TurboID and GFP-Rab7, COR1C-GFP, or COR1C ACT–-ΔCC-GFP. 16 h after transfection, cells were incubated in 500 μM Biotin (B4501-500 MG; Sigma-Aldrich) for 3 h followed by two 1× PBS washes to remove excess biotin. Cells were then trypsinized and pelleted, and the pellet was washed twice with ice-cold 1× PBS. Cells were then lysed in 250 μl freshly made lysis buffer + PIC (50 mM Tris, pH 7.5, 150 mM NaCl, 10% glycerol, 1% NP-40 [for Cyto-V5-TurboID NP40 was replaced with TX-100 due to problems with availability], 1× Calbiochem protease inhibitor cocktail III EDTA free) nutating at 4°C for 1 h. Lysed cells were spun at 16,100 *g* for 10 min at 4°C to pellet insoluble cell debris. 5% of the lysis volume was collected as “load” sample. The remaining sample was loaded on to biotin antibody agarose beads (ICP0615; Immunechem) and left nutating overnight at 4°C. Bead-bound samples were washed twice for 5 min on a nutator at 4°C with cold high salt wash (50 mM Tris, pH 7.5, 1 M NaCl, 1 mM EDTA, 1% NP40, 0.1% SDS, 0.5% sodium deoxycholate [Sawyer et al., 2019]) followed by 4× cold lysis buffer without PIC washes at 4°C. Proteins were eluted off beads with 2× Laemmli sample buffer (with 355 mM 2-mercaptoethanol) and run on 4–12% Criterion TGX gels. Using standard immunoblotting protocols V5 (turboID) and GFP (potential interactors) were blotted for. To quantify pulldown, elute and input signals were background subtracted using local background of the band and then elute signal was divided by input signal.

### ARP2/3 inhibition by CK-666 treatment

COS-7 cells were prepared for imaging as detailed in the transfection protocol. Cells with extended actin structures were identified and imaged. Cells were treated with 150 μM CK-666 (182515-25MG; Millipore Sigma) in order to ensure fast uniform ARP2/3 inhibition. This was critical to ensure confidence in tracking the same endosome bud before and after drug treatment. Cells were imaged 30 s after addition of CK-666.

### CI-M6PR sorting

The protocol was performed as previously described in Dong et al. (2016); Hoyer et al. (2018). Briefly, COS-7 cells were prepared for imaging as detailed in the transfection/KD protocols. Cells were incubated for 1 h with anti CI-M6PR antibody 1:1,000 in SF DMEM before being fixed with warm 4% sucrose 4% PFA in 1× PBS. Cells were stained with anti-Giantin antibody 1/500 to mark the Golgi. To measure retrograde recycling, a 5 × 5–μm ROI was placed over the anti-Giantin signal, and CI-M6PR signal was measured yielding a “sorted Golgi” signal. All signal outside the Golgi ROI and within the cell was measured as “unsorted vesicular” signal. Background was measured outside of the cell via another 5 × 5–μm ROI and subtracted from the Golgi and vesicular signals. A ratio of background-corrected vesicular to Golgi signal was then calculated to yield a final sorting score.

### ER contact quantification

ER contact at FAM21-positive endosome buds was adapted from Hoyer et al. (2018). Briefly, COS-7 cells were transfected with GFP-Rab7, mCh-FAM21, BFP-Sec61β, COR1A/1B/1C siRNA, and rescued with either SiRES COR1C-Halo, SiRES COR1C ACT–-Halo, SiRES COR1C ΔCC-Halo, SiRES COR1C ACT–-ΔCC-Halo, or SiRES COR1C CC-Halo. Time lapses were collected at 2-s intervals for 2 min. We tracked every FAM21-positive endosome bud that was stable (did not collapse or undergo fission) for ≥30 consecutive frames in regions of the ER that were clearly resolvable. For each frame, overlap of the ER with the FAM21-positive endosome bud was assessed. This was used to calculate a percentage of time each bud was overlapping with the ER. Bud percentage contact was then averaged per cell to give an ER endosome bud contact score per cell.

### Statistics

All data described are from at least three biological replicates. All data were graphed as box-and-whisker plots with median (indicated by line) and mean (indicated by X) shown. When comparing two samples, two-tailed Student’s *t* tests were used. For comparisons across multiple samples, significance testing was done by first establishing that significant differences exist between conditions by one way ANOVA. If variation between conditions was significantly greater then within conditions, this was followed by post hoc significance testing with Tukey’s test. *, P < 0.05; **, P < 0.01; ***, P < 0.001. Analyses were all performed using GraphPad Prism 8. Details of significance calculations as well as *n* values for each quantification are reported in the relevant figure legends.

### Online supplemental material

Fig. S1 shows immunoblots of type I coronin depletion in COS-7 cells, demonstrating siRNA efficacy. Fig. S2 shows immunoblot of type I coronin depletion and rescue in COS-7 cells, demonstrating depletion and rescue efficacy. Fig. S3 shows TurboID controls, indicating that ARP3 TurboID activity is specific. Fig. S4 shows graphs of vacuole diameter and bud length, indicating they are not changed significantly in depletion and rescue experiments. Fig. S5 shows immunoblot of FAM21 depletion and type I coronin depletion and rescue in COS-7 cells for CI-M6PR sorting. Video 1 shows the FAM21 (green) LE (magenta) fission event from Fig. 1 A. Video 2 shows the ARP3 (green) LE (magenta) fission event from Fig. 1 D. Video 3 shows the actin (green) LE (magenta) fission event from Fig. 1 G. Video 4 shows the COR1C (green) LE (magenta) fission event from Fig. 1 J. Video 5 shows actin (green) localization on LE bud (gray) in control siRNA-treated cell, related to Fig. 2, A and B. Video 6 shows actin (green) localization on LE bud (gray) in COR1A/1B/1C-depleted cell, related to Fig. 2 B. Table S1 lists primers.

## Supplementary Material

Table S1 lists primers.

SourceData F4 is the source file for Fig. 4.

SourceData FS1 is the source file for Fig. S1.

SourceData FS2 is the source file for Fig. S2.

SourceData FS3 is the source file for Fig. S3.

SourceData FS5 is the source file for Fig. S5.
