# Supplementary figures and images for: Coronin 1C restricts endosomal branched actin to organize ER contact and endosome fission
Source: J Cell Biol. 2022 Jul 8;221(8):e202110089. doi: 10.1083/jcb.202110089 (PMC9274145; doi:10.1083/jcb.202110089)

|                    |   |   |   |   |   |   |   |   |   |   |   |   |                    |
|--------------------|---|---|---|---|---|---|---|---|---|---|---|---|--------------------|
| ARP3-V5-TurboID    | ● | ● | ● | ● | ● | ● | ● | ● | ● | ● | ● | ● | ARP3-V5-TurboID    |
| GFP-Rab7           | ● | ○ | ○ | ● | ○ | ○ | ● | ○ | ○ | ● | ○ | ○ | GFP-Rab7           |
| COR1C-GFP          | ○ | ● | ○ | ○ | ● | ○ | ○ | ● | ○ | ○ | ● | ○ | COR1C-GFP          |
| COR1C ACT- ΔCC-GFP | ○ | ○ | ● | ○ | ○ | ● | ○ | ○ | ● | ○ | ○ | ● | COR1C ACT- ΔCC-GFP |

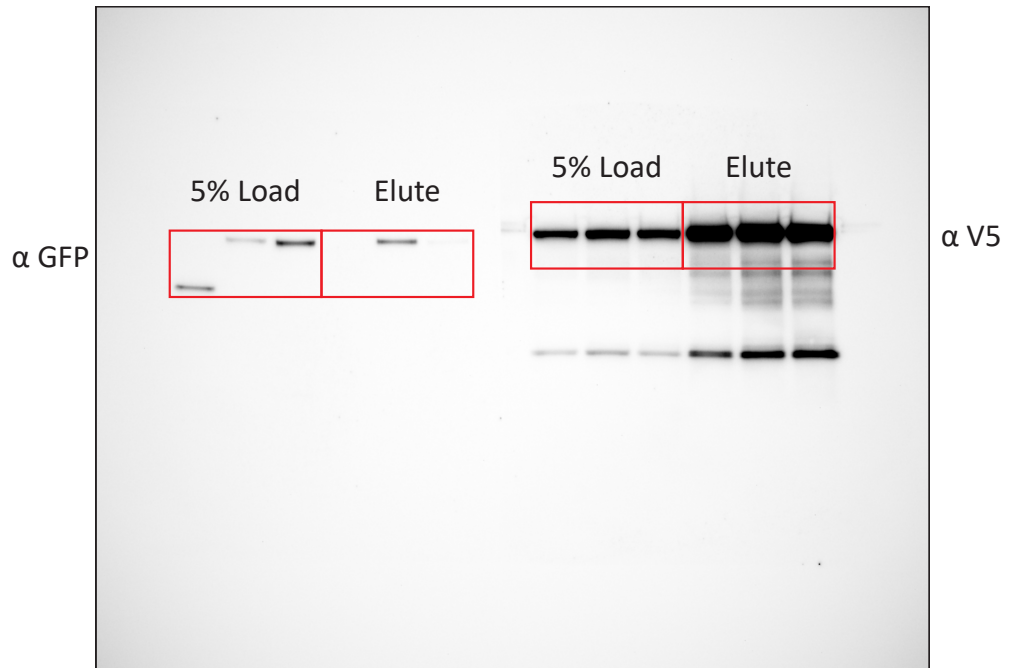

Supplement: SourceData F4 — is the source file for Fig. 4. [file JCB_202110089_SourceDataF4.pdf]

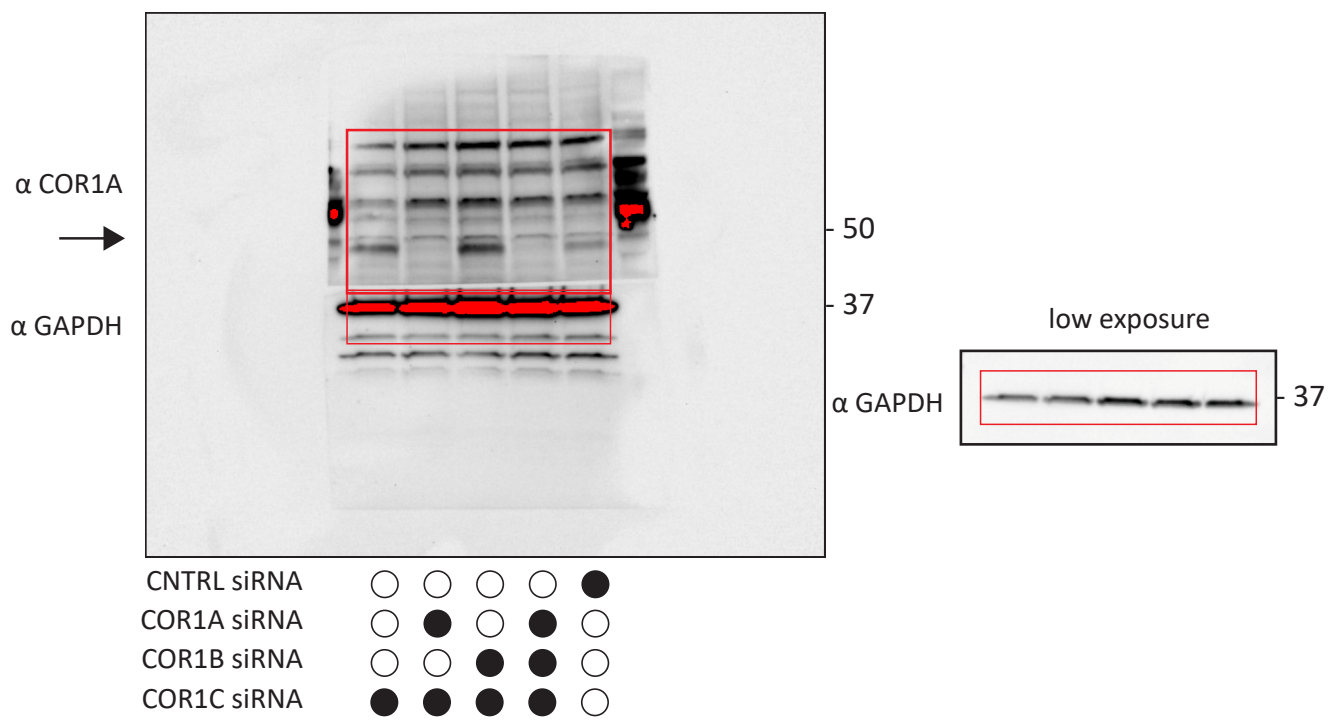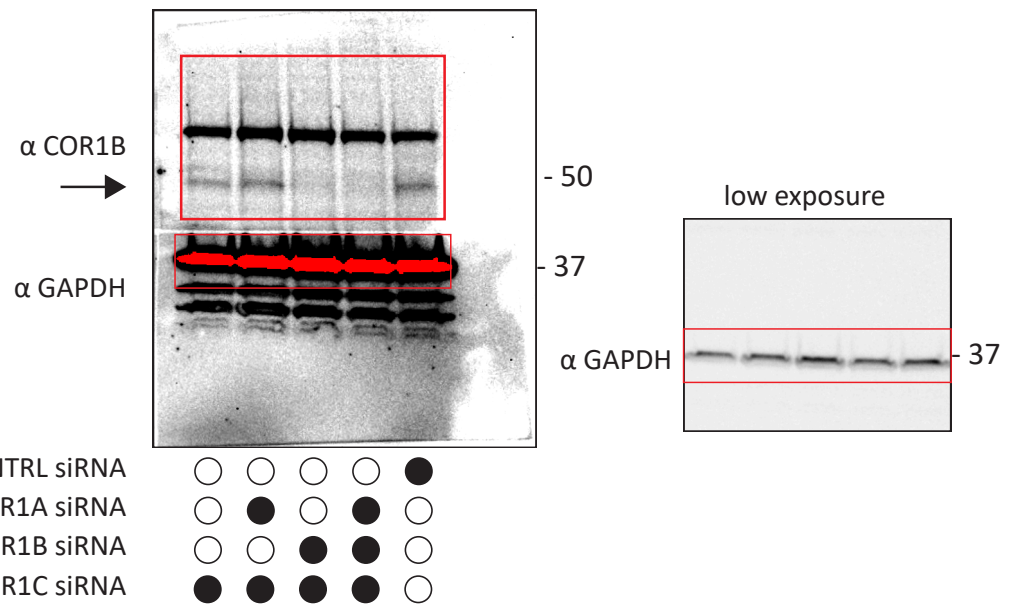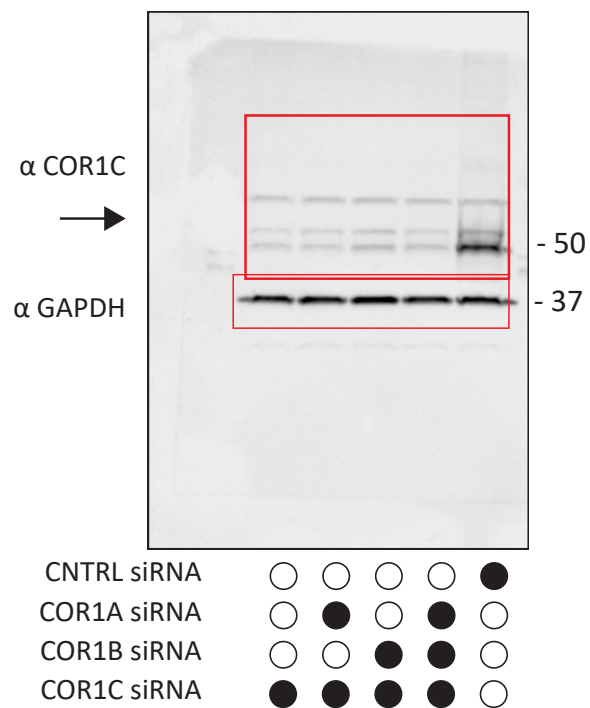

Supplement: SourceData FS1 — is the source file for Fig. S1. [file JCB_202110089_SourceDataFS1.pdf]

# A

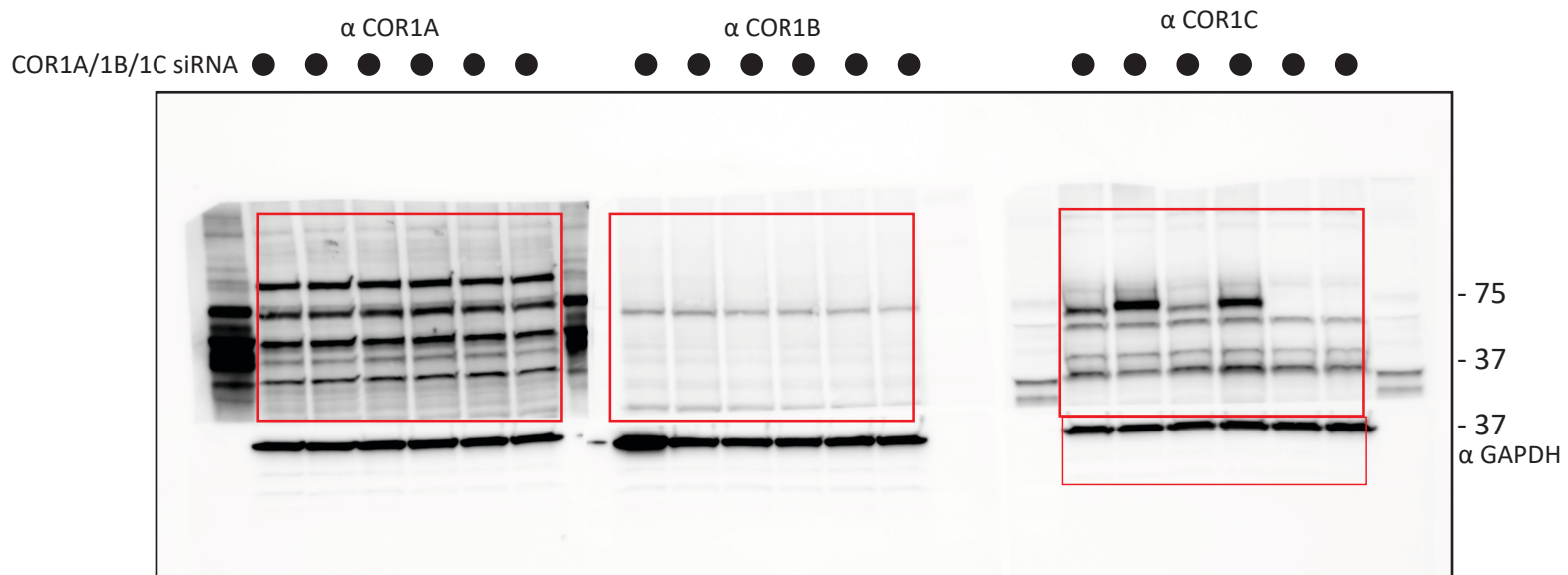

# B

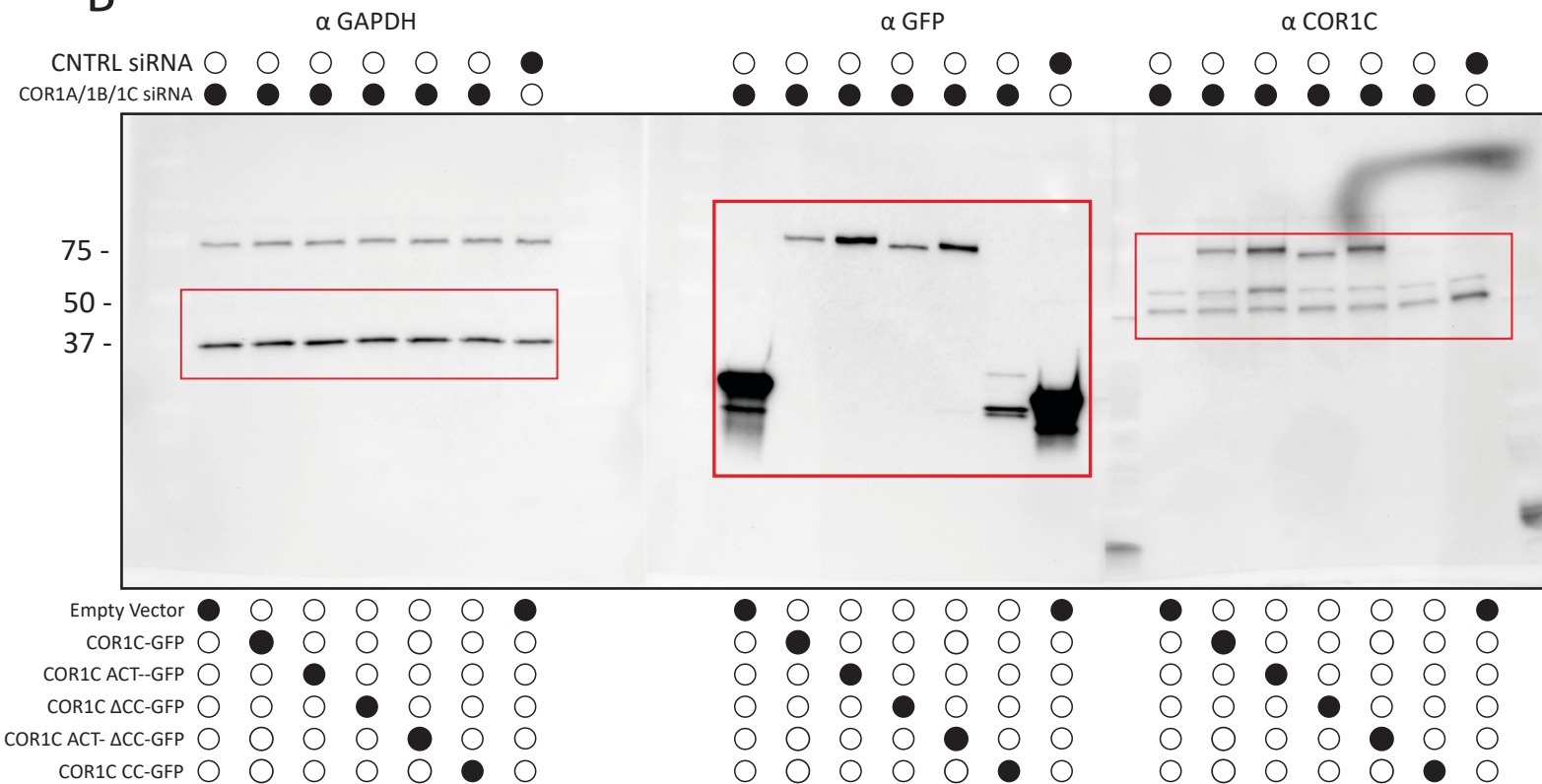

Supplement: SourceData FS2 — is the source file for Fig. S2. [file JCB_202110089_SourceDataFS2.pdf]

A

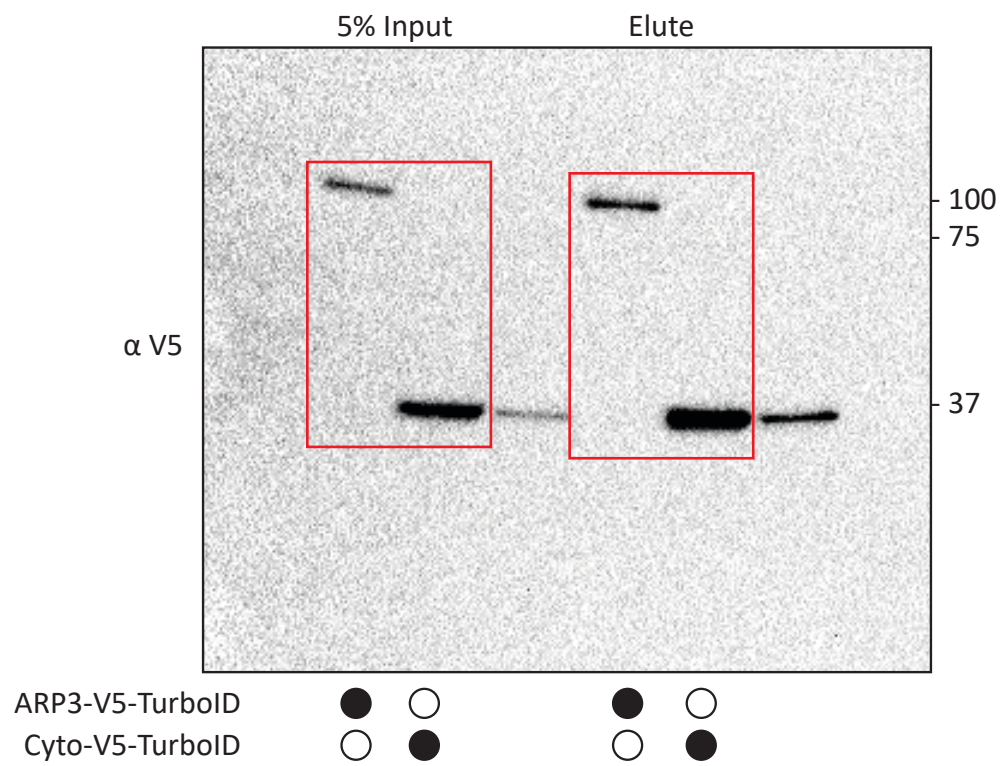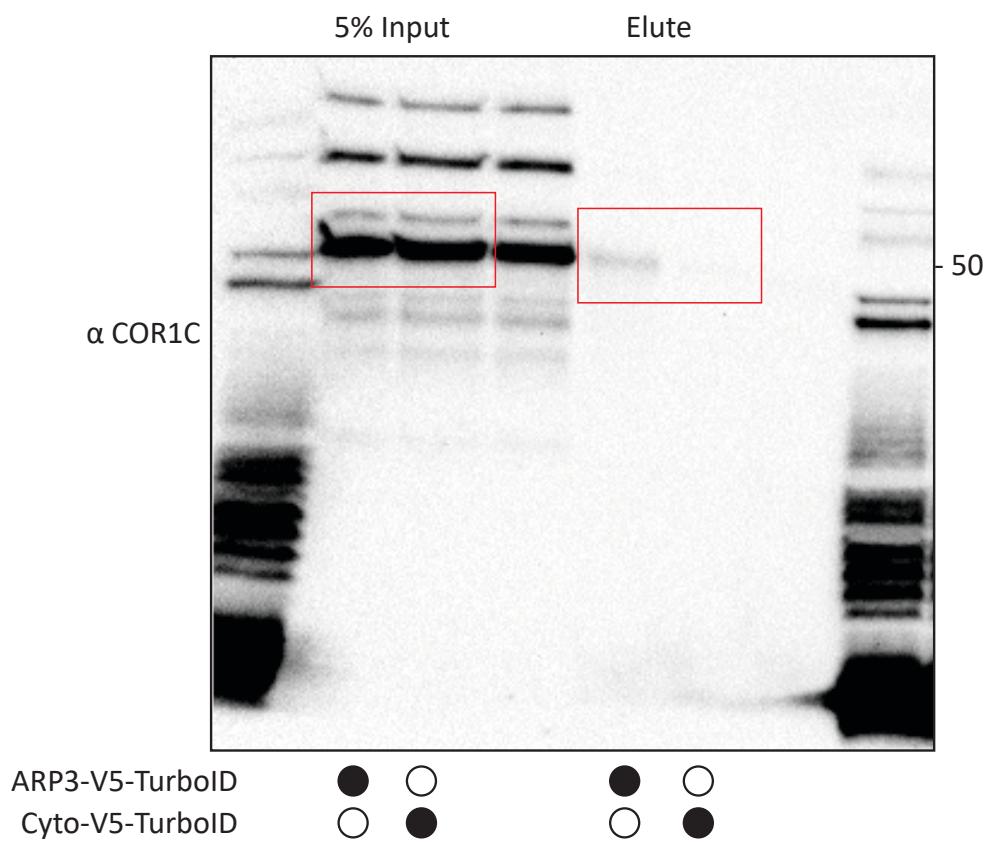

C

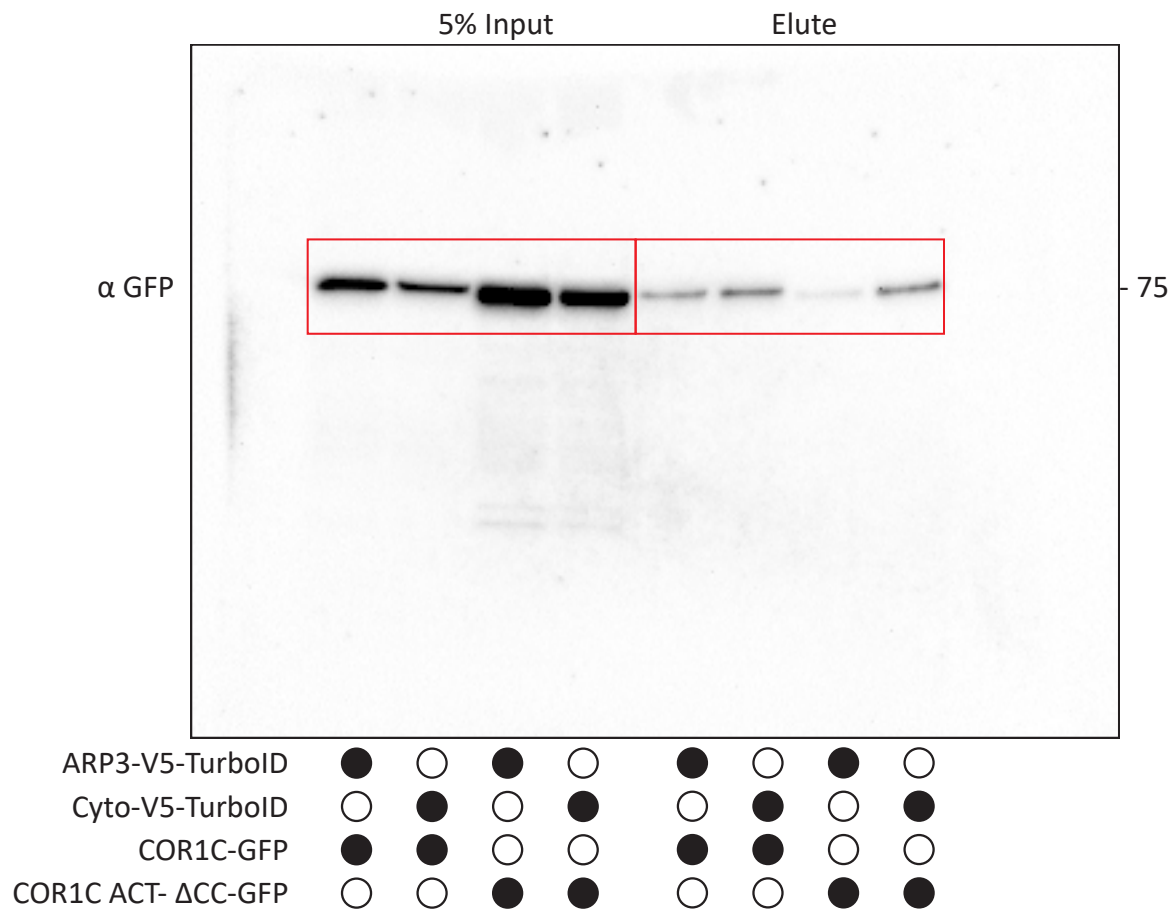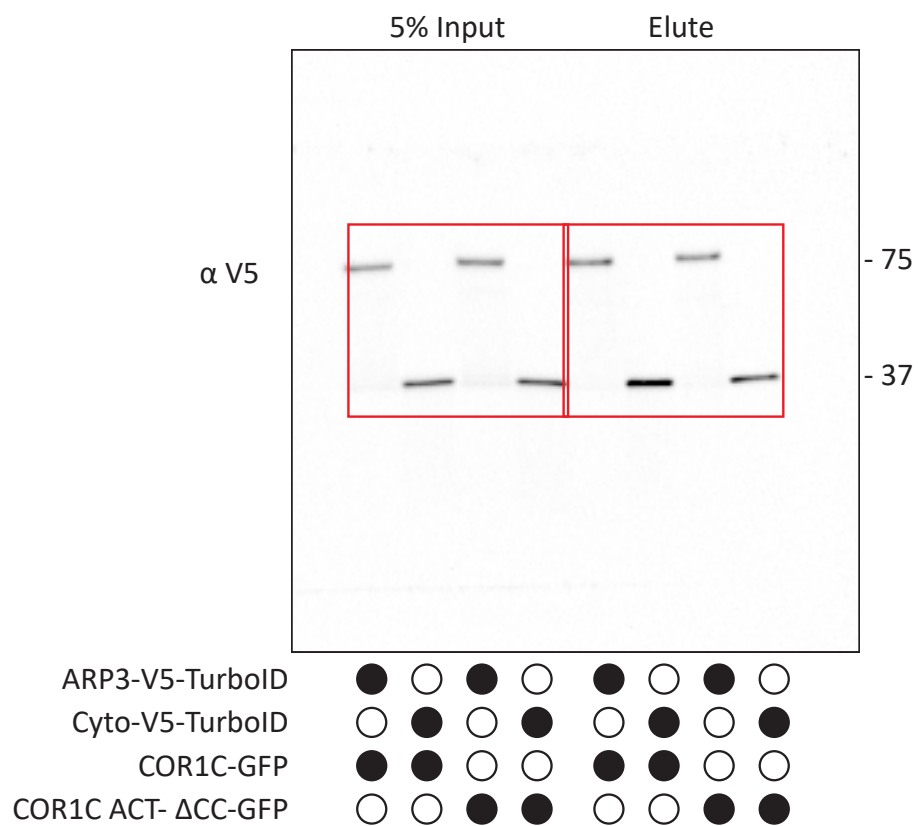

Supplement: SourceData FS3 — is the source file for Fig. S3. [file JCB_202110089_SourceDataFS3.pdf]
