# Supplementary material for: Coronin 1C restricts endosomal branched actin to organize ER contact and endosome fission
Source: J Cell Biol. 2022 Jul 8;221(8):e202110089. doi: 10.1083/jcb.202110089 (PMC9274145; doi:10.1083/jcb.202110089)
Supplement: SourceData FS5 — is the source file for Fig. S5. [file JCB_202110089_SourceDataFS5.pdf]

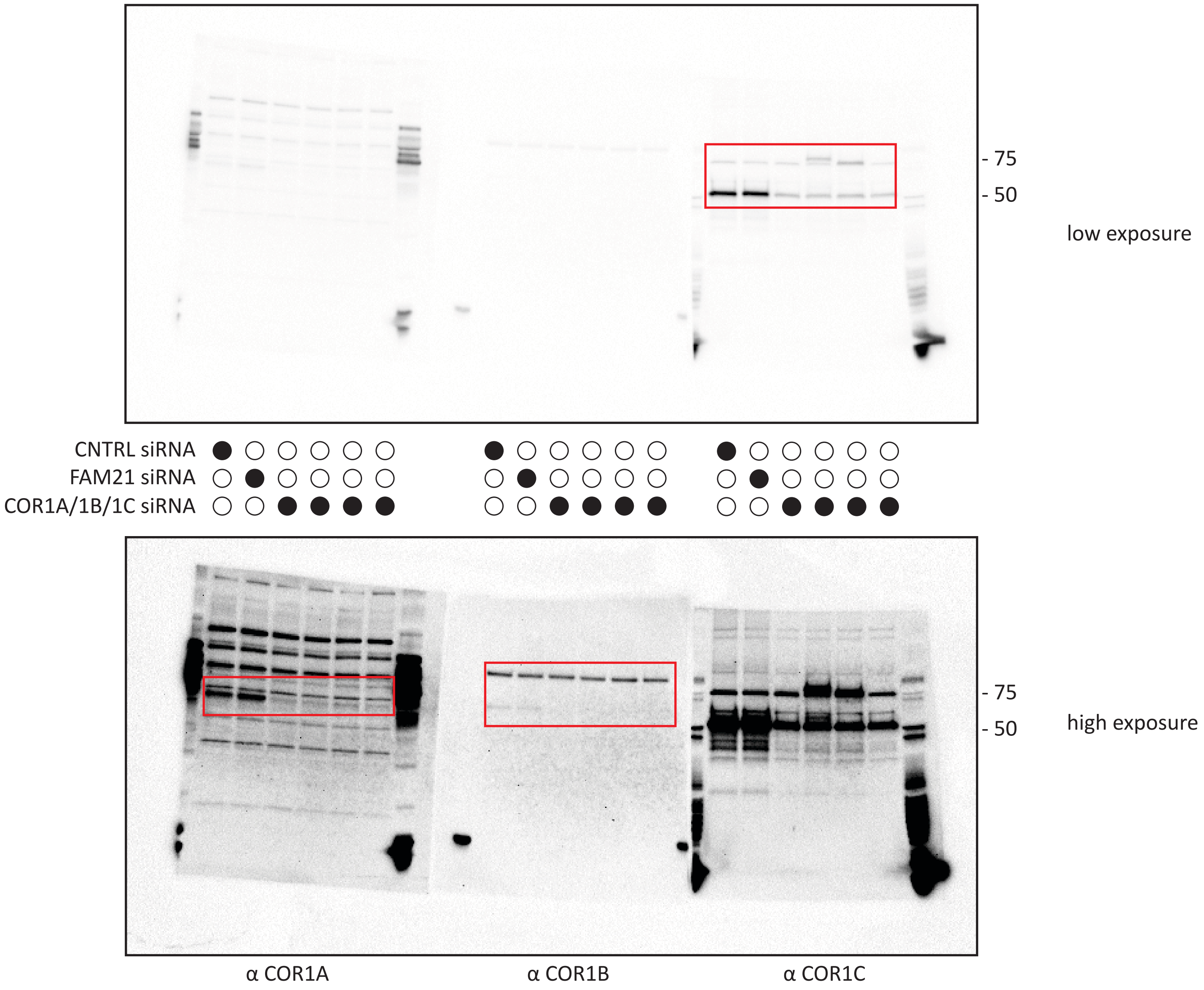
- 75
- 50
low exposure
CNTRL siRNA
FAM21 siRNA
COR1A/1B/1C siRNA
- 75
- 50
high exposure
α COR1A
α COR1B
α COR1C

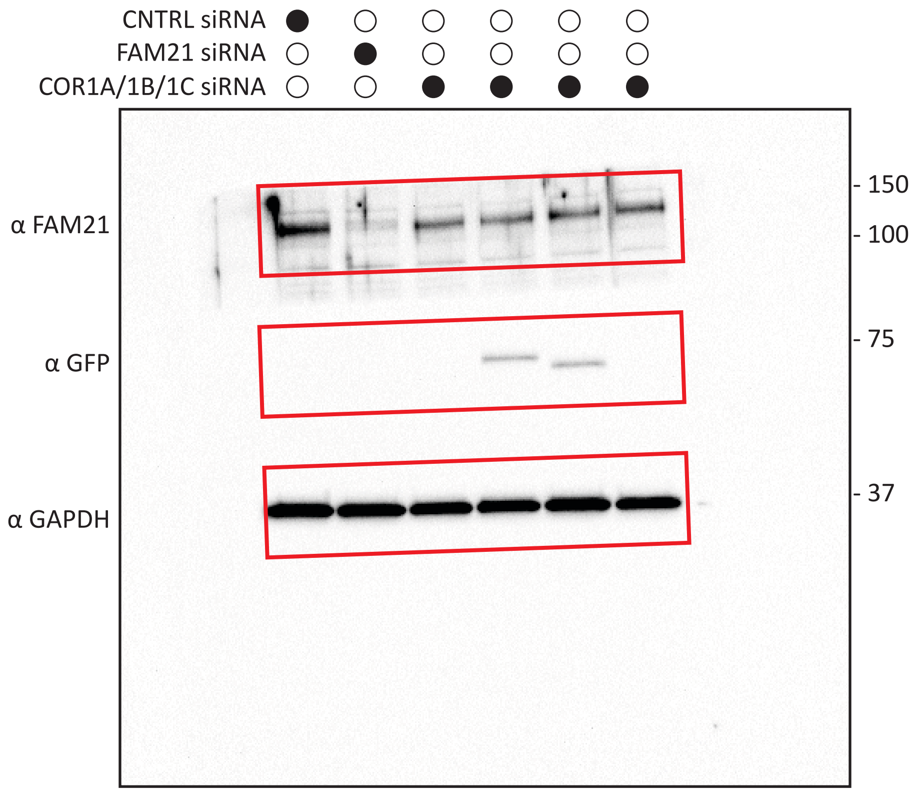
CNTRL siRNA
FAM21 siRNA
COR1A/1B/1C siRNA
α FAM21
α GFP
α GAPDH
- 150
- 100
- 75
- 37
